# Mapping small inland wetlands in the South-Kivu province by integrating optical and SAR data with statistical models for accurate distribution assessment

**DOI:** 10.1038/s41598-023-43292-7

**Published:** 2023-10-17

**Authors:** Chuma B. Géant, Mushagalusa N. Gustave, Serge Schmitz

**Affiliations:** 1 Faculty of Agriculture and Environmental Sciences, Université Evangélique en Afrique (UEA), P.O Box: 3323, Bukavu, Democratic Republic of the Congo; 2 Department of Geography, University of Liège, UR SPHERES-Laplec, Bât. B11, Quartier Village 4, Clos Mercator 3, Liège, Belgium

**Keywords:** Environmental sciences, Ecological modelling, Ecosystem ecology, Freshwater ecology, Riparian ecology, Wetlands ecology

## Abstract

There are several techniques for mapping wetlands. In this study, we examined four statistical models to assess the potential distribution of wetlands in the South-Kivu province by combining optical and SAR images. The approach involved integrating topographic, hydrological, and vegetation indices into the four most used classifiers, namely Artificial Neural Network (ANN), Random Forest (RF), Boosted Regression Tree (BRT), and Maximum Entropy (MaxEnt). A wetland distribution map was generated and classified into 'wetland' and 'non-wetland.' The results showed variations in predictions among the different models. RF exhibited the most accurate predictions, achieving an overall classification accuracy of 95.67% and AUC and TSS values of 82.4%. Integrating SAR data improved accuracy and precision, particularly for mapping small inland wetlands. Our estimations indicate that wetlands cover approximately 13.5% (898,690 ha) of the entire province. BRT estimated wetland areas to be ~ 16% (1,106,080 ha), while ANN estimated ~ 14% (967,820 ha), MaxEnt ~ 15% (1,036,950 ha), and RF approximately ~ 10% (691,300 ha). The distribution of these areas varied across different territories, with higher values observed in Mwenga, Shabunda, and Fizi. Many of these areas are permanently flooded, while others experience seasonal inundation. Through digitization, the delineation process revealed variations in wetland areas, ranging from tens to thousands of hectares. The geographical distribution of wetlands generated in this study will serve as an essential reference for future investigations and pave the way for further research on characterizing and categorizing these areas.

## Introduction

Wetlands are highly abundant habitats within the Congo basin, particularly in the Democratic Republic of the Congo (DRC) and its eastern provinces^1–4^. These wetlands play a crucial role in the South-Kivu province, providing goods and services to the local community while supporting biodiversity. With their diverse range of uses and significant agricultural potential, it is imperative to pay special attention to the conservation and management of these ecosystems^5,6^. Although the importance of wetland ecosystem services is widely recognized, more detailed inventories are needed to ensure the effective implementation of conservation strategies. Many wetlands in the region still need to be identified and are not represented on publicly available maps. This knowledge gap poses challenges to their conservation and sustainable use. More definition are available for wetland, the one proposed by Amler et al.^7^ was adopted: wetland in the East African landscape refers to “a diverse and dynamic ecosystem characterized by the presence of water, both permanent and seasonal, along with distinct ecological features, including habitats such as marshes, swamps, floodplains, inland valleys, coastal, mangroves, and shallow lakes, with varying water depths (most lower than 1 m) and vegetation types”.

The vast geographic expanse and complex distribution of wetlands in eastern DRC present significant challenges for conducting comprehensive inventories^1,2^. However, recent advancements in technology, such as the availability of high-resolution georeferenced field data archives and open access to high-spatial-resolution remote sensing data, coupled with the application of artificial intelligence (AI) techniques, have opened up new possibilities for accurate and detailed wetland mapping^8–11^. Despite the importance of wetlands, there still needs to be more knowledge regarding their distribution and status. Closing this knowledge gap requires assessing the potential distribution and characterizing wetlands at national and provincial levels. Effective management and monitoring methods are essential for conserving and protecting wetlands, as these ecosystems face multiple pressures from human activities, invasive species, and climate change. The loss or degradation of wetlands significantly impacts their ability to sustain biodiversity, maintain water quality, mitigate floods, and sequester carbon^5,6^. Accurate mapping of wetlands with high spatial and thematic precision plays a crucial role in their effective management and monitoring. These maps help identify potential risks and pressures on wetlands and assess the effectiveness of wetland conservation programs^11,12^.

The initial studies on wetland mapping in the Democratic Republic of the Congo (DRC) date back to 2010, with researchers such as Bwangoy et al.^1,2^; and Lee et al.^13^ exploring various aspects of wetland classification and monitoring. To gather data, these studies used optical sensors, specifically Landsat 5 MSS and 7 TM. However, the integration of Synthetic Aperture Radar (SAR) imagery was also incorporated due to its ability to penetrate through vegetation canopies and its sensitivity to moisture conditions. For SAR data, the PALSAR radar and SRTM datasets were utilized. Bwangoy et al.^13^ demonstrated that the integration of optical and SAR data resulted in high accuracy levels, surpassing existing maps such as Africover (77%) and The JRC/GRFM Regional Flooded Forest Map of Central Africa (73.0%), with a Kappa coefficient exceeding 0.70. Utilizing this approach, Bwangoy et al.^13^ estimated the coverage of wetlands in the DRC to be approximately 440,000 km^2^, accounting for 19.2% of the country's total area.

The eastern region of the Democratic Republic of the Congo (DRC) and eastern Africa as a whole showcase a diverse range of landscapes^7,14^. However, small inland wetlands, characterized by their size (< 500 ha), often go unnoticed and receive limited attention in conservation and restoration efforts, mainly due to the challenges associated with identifying them within large regions. Many of these wetlands exhibit seasonal variations in water levels and vegetation, making remote sensing a valuable tool for their detection. Despite their potential for agricultural production and various uses in South-Kivu province and eastern DRC, many of these wetlands remain undocumented on official maps. The lack of official recognition has resulted in their unsustainable exploitation.

While other regions in Africa employed classic mapping methods like supervised or unsupervised classification, including Maximum Likelihood, ISODATA, PCA, or K-means, the DRC utilized a "decision tree" model for wetland mapping and identifying emerging wetland forests^2^. However, the limitations of the satellite images used, characterized by low spatial and spectral resolutions, hindered the production of maps suitable for provincial or territorial decision-making. Consequently, these studies have yet to achieve results at such scales. Nevertheless, these studies served as a foundation for subsequent mapping efforts at the national level.

The combination of optical spectral and SAR indices has proven to be suitable for wetland mapping, as indicated by studies conducted by Kulawardhana et al.^14^, García and Lleellish^15^; Farda et al.^16^; Alves et al.^17^; Sun et al.^18^; López-Tapia et al.^19^; Islam et al.^20^; Saha et al.^21^; and Pham et al.^10^ to mention just a few. For decades, Synthetic Aperture Radar (SAR) are currently integrated for flood process, wetlands mapping, and vegetation monitoring^22–27^. SAR data is particularly beneficial for wetland mapping because it can penetrate vegetation canopies (depending on the wavelength) to identify inundation and is sensitive to moisture conditions. While initial wetland mapping research predominantly relied on optical satellite images, SAR sensors offer the advantage of acquiring data even in the presence of clouds, haze, and other atmospheric disturbances, as they emit their own incident radiation^28^. However, weather conditions like wind, rain, and cold temperatures can impact SAR data quality. Additionally, the integration of multi-sensor images allows for the consideration of both water and vegetation factors, which can influence wetland-mapping accuracy.

Among the various methods used for mapping and delineating wetlands, those incorporating topographic features, hydrological processes, and vegetation aspects tend to offer high accuracy. These approaches consider all three essential factors in wetland definition^7,29^. Similar methodologies have been tested in different regions, including Canada^30,31^, Nigeria^32^, South Africa^33^, and other areas^34^, with varying levels of accuracy depending on the image and model types used. Classifiers such as Decision Tree (DT), Support Vector Machine (SVM), Artificial Neural Network (ANN), Logistic Regression Model (LRM), and Maximum Entropy (Maxent) have been utilized, with Random Forest (RF) and SVM showing promising results in terms of accuracy. These models have also been tested for wetland distribution and other applications, proving effective^35,36^. Overall, both DT, SVM, ANN, RF, BRT, KNN classifiers are the most famous worldwide classification algorithms used for wetland mapping^37^.

Despite abundant data and tools, there still needs to be more knowledge regarding which models can accurately assess the practical distribution map of small inland wetlands and delineate them. Debates persist regarding the choice of models and the types of images or indices to employ for this purpose. However, advancements in technology have significantly contributed to the field of Geographic Information Systems (GIS) and Remote Sensing (RS), enabling the modeling and prediction of wetland ecosystems at both small and macro scales, as well as the assessment of distribution factors. Modern geo-statistics and techniques integrated into GIS tools facilitate efficient modeling of wetland distribution, while RS provides valuable imagery for detecting, digitizing, and estimating wetland distribution. These advancements have enhanced our understanding and capability to assess wetland distribution accurately, contributing to effective wetland management and conservation efforts.

To address the need for identifying and delineating these low-lying wetlands in the eastern region of the DRC, we propose an approach that combines optical images from the Sentinel satellites with Synthetic Aperture Radar (SAR) images. The methodology draws upon previous research by Mwita et al.^30,38,39^, and Garba et al.^32^, with modifications such as replacing Landsat images with Sentinel (1 and 2) and ALOSPALSAR. Additionally, we evaluate the performance of four widely used statistical classifiers in wetland mapping.

These statistical models have been extensively studied and proven to enhance the accuracy of wetland distribution predictions^40,41^. Over the past decade, these techniques have garnered significant attention in ecosystem modeling and forecasting thanks to their ability to improve predictive capabilities. One key advantage of these mathematical models is their utilization of different types of independent and dependent variables, including categorical and quantitative variables. This versatility extends their applicability beyond wetland mapping and serves as a valuable reference for researchers in various fields of science, such as biology, sociology, and agronomy.

### Study objectives

The overall objective of this study is to contribute to the identification and study of wetlands in the Democratic Republic of the Congo (DRC) by developing improved methods for mapping small inland wetlands. Specifically, this study aims to achieve the following objectives: (1) Identify the critical explanatory variables derived from remote sensing data, including Sentinel-1 and Sentinel-2, as well as ALSOPALSAR, and field data, that are relevant for modeling the distribution of small wetlands in eastern DRC; (2) Evaluate the capabilities of single-date Sentinel optical data and their combination with Synthetic Aperture Radar (SAR) data for mapping small wetlands in the South-Kivu province.

This evaluation will include assessing the accuracy of these mapping methods and identifying potential errors; (3) Digitize and characterize the identified wetlands, including analyzing their morphological characteristics such as area and perimeter; (4) Discuss the strengths and limitations of the mapping methods employed in this study, providing an overview of the advantages and challenges associated with integrating optical, topographic, and SAR indices, as well as using novel classifiers, to achieve accurate mapping results.

To address these objectives, it is hypothesized that integrating optical, topographic, and SAR indices with new classifiers will result in a more accurate method for mapping small wetlands. Additionally, it is suggested that fully polarimetric SAR imagery can provide valuable information about surface scattering mechanisms, allowing for a more precise distinction of small wetlands. Furthermore, including SAR data and novel vegetation indices is expected to improve the mapping process. Finally, it is anticipated that the delineation of wetlands after digitization will reveal various areas with distinct morphological characteristics. By addressing these research hypotheses, this study will enhance our understanding of wetland mapping methodologies in the context of the DRC, with a specific focus on small wetlands.

## Methods

### An overview of the study area

**Figure 1:**
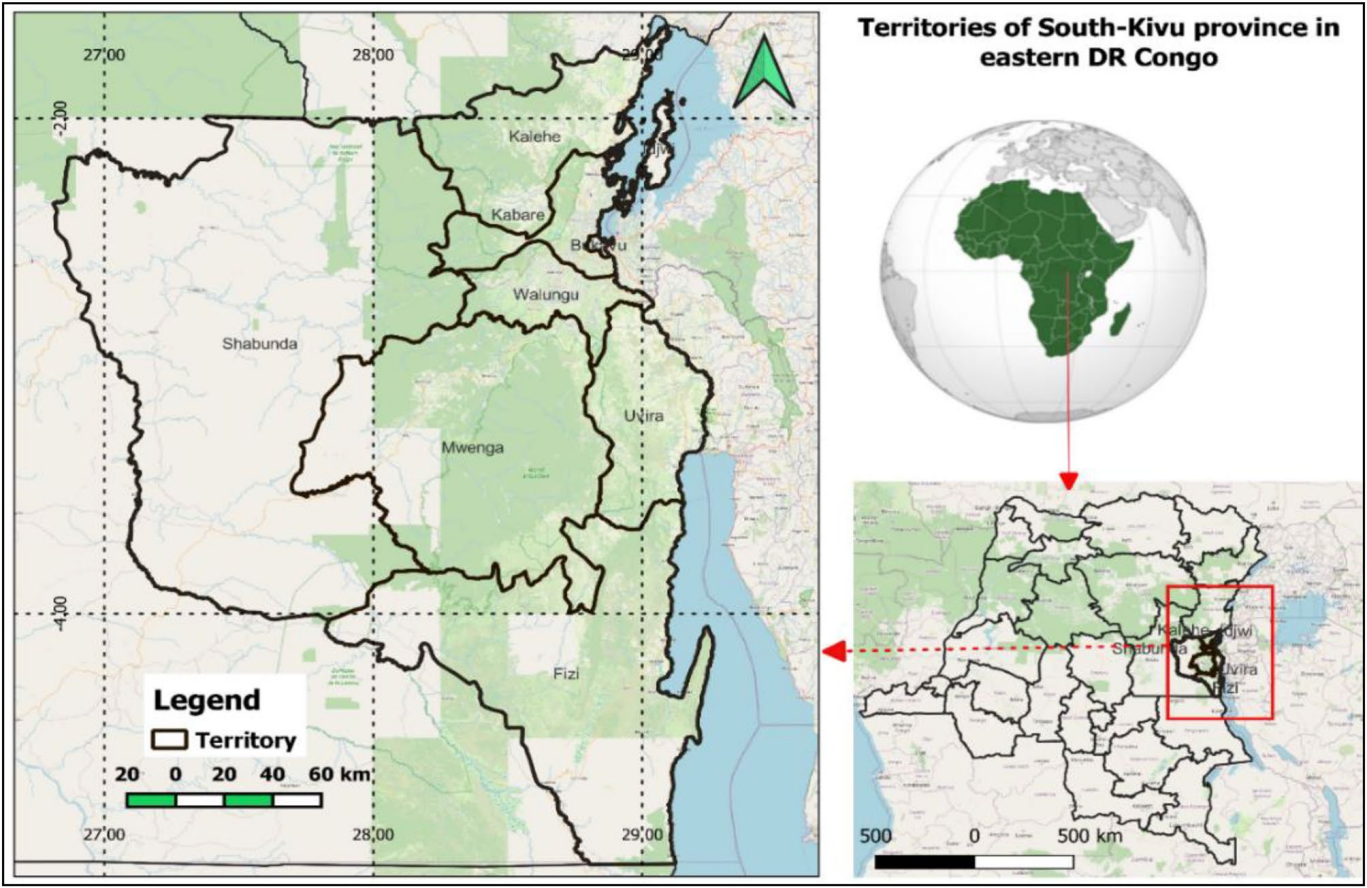
Territories of the South-Kivu province in Eastern DR Congo (map created using ArcGIS 10.7 Esri-TM: http://www.esri.com).

The South-Kivu province is situated in eastern DRC and shares borders with Rwanda, Burundi, and Tanzania. Covering an area of approximately 64,791 km^2^, it is one of the 26 provinces of the Democratic Republic of the Congo. South-Kivu accounts for around 2.73% of the total land area of the country^42,43^. The province is home to approximately 6.2 million people, with 47% of the population residing in rural areas^44^. Geographically, the province is located between latitudes 1.5836° and 5.0103° South and longitudes 26.8106° and 29.3890° East (Fig. 1).

**Figure 2:**
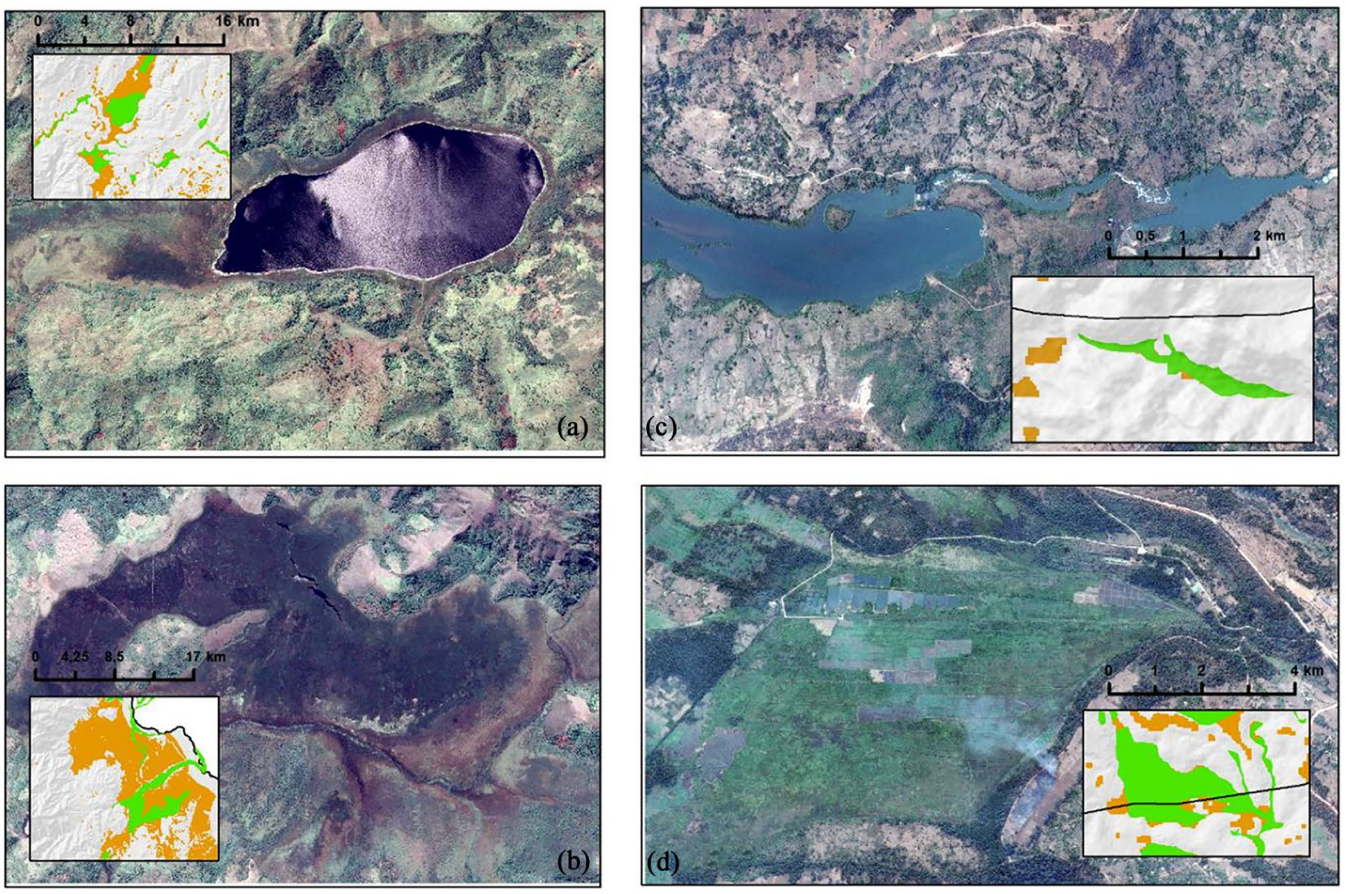
Diversity of wetlands in South-Kivu, Eastern DR Congo (**a)** very high altitude lake Lubwe in Itombwe, (**b)** peatland in Lugana, (**c**) coastal of the Ruzizi river, and (**d**) flooded plain for rice production in Ruzizi plain. map created using ArcGIS 10.7 Esri-TM: http://www.esri.com.

The province is divided into eight territories, namely (Shabunda, Kalehe, Uvira, Walungu, Kabare, Idjwi, Mwenga, and Fizi) including the city of Bukavu (Fig. 1), and each territory in municipalities referred to as "Groupement". The province has a variety of landscapes ranging from low to very high altitudes. The elevation fluctuates from ~ 512 to 3464 m above the sea level (m.a.s.l) and decreases from east to west. The Congolese "Cuvette Centrale" begins in the Shabunda and Mwenga territories, whereas reliefs and valleys, including mountains and the Mitumba Chain Mountains, characterize the eastern and central territories. On the left, the Ruzizi plain is a broad plain extending into the Fizi territory, in the territory of Walungu and Uvira, as well as the highland favorable to the growth of a diverse type of wetlands and especially swamps, marshes, and peats^44^. Various wetlands occur due to these physical conditions (Figs. 2 and 4).

South-Kivu province is characterized by a humid tropical and equatorial climate. According to Balasha et al.^6^, the mean annual rainfall of ~ 1500–1800 mm each year and an average annual temperature varies between 11° and 25 °C (Supplementary material S1) are observed in the province. Seven primary soil categories predominate in the South-Kivu mainly Haplic Acrisols, Humic Cambisols, Humic Ferrasol, Luvic Phaeozems, Mollic Fluvisols, Vertisols, and Gleyic Solonchaks^45,46^. Histisols (organic soil) are observed in some territories in small areas. The peat is used as energy fire material for food cooking in small quantities using small bricks. These bricks can replace a sizable quantity of charcoal or wood. In the South Kivu, peat is hand-harvested in the Kakonda peat (in Kabare), Hogola (at Nyangezi) in the Chiherano, and Kachandja peats (in Walungu). The hydrography is extensive and thick, with several small sources, large rivers (such as the Ruzizi, Elila, Ulindi, Itombwe, and Lwama), as well as lakes. Additionally, the province is a part of the "African Great Lakes" region. It has Lake Kivu, Tanganyika, and a few more small lakes and ponds (such as Lubwe in Itombwe). The vegetation comprises highland forests, herbaceous savanna, wooded swamps, and dense forest.

The land cover map was taken from the 2018 version of the European Space Agency's (ESA) Climate Change Initiative Land Cover Project (CCI-LC) (cds.climate.copernicus.eu) product. The CCI-LC identified 21 land cover classes in South-Kivu (Supplementary material S2), which can initially be categorized using the FAO's land cover classification system (LCCS). The province has protected areas, mainly the Kahuzi-Biega National Park (KBNP), the Natural Reserve of Itombwe (NRI), Maniema, South-Masisi, the Mont Kabobo (National Park of Ngamikka), and the Luama-Kivu Hunting Reserve^47^.

### Materials and methods

**Figure 3:**
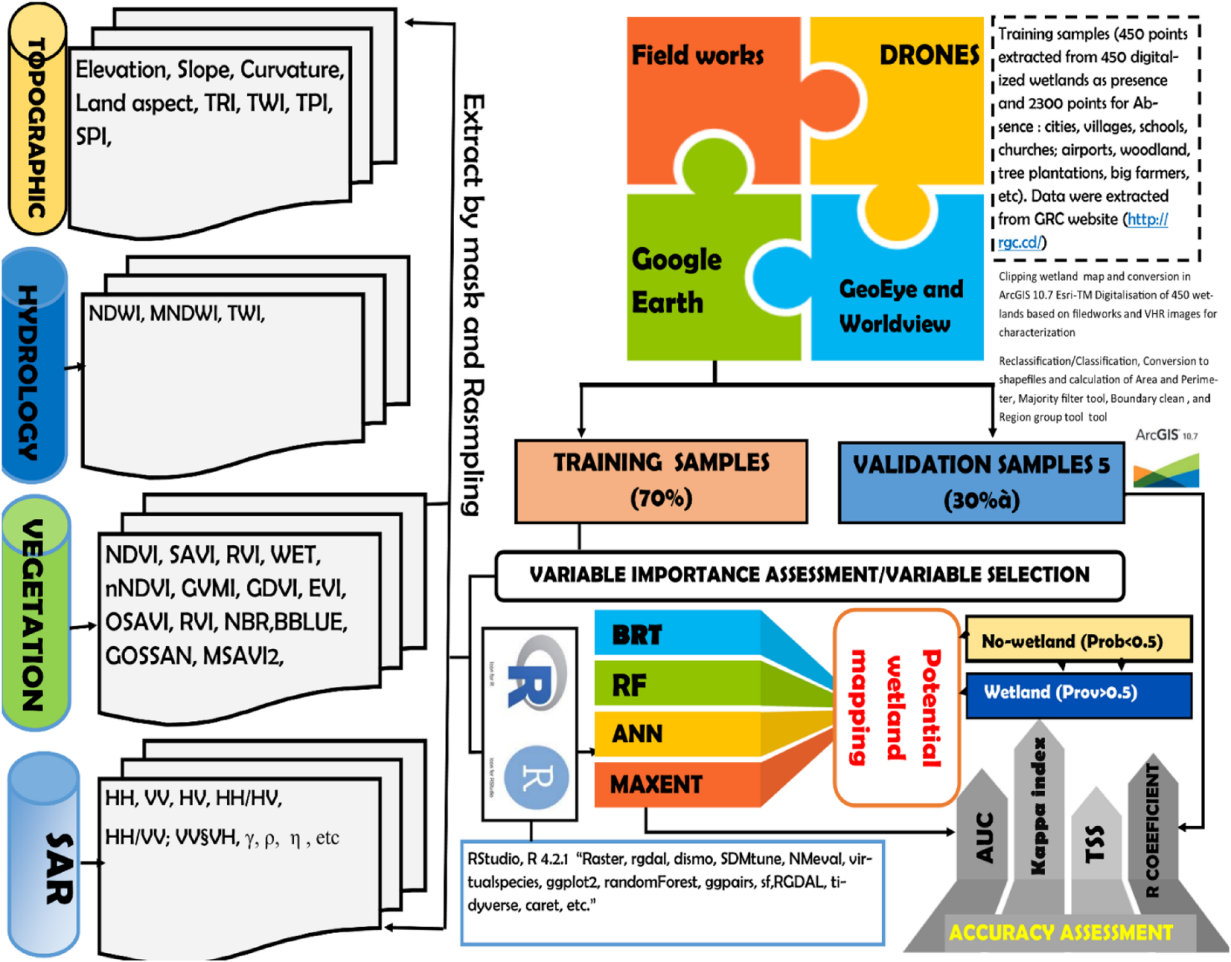
Flowchart of the methods used to map wetlands in South-Kivu by using optical, SAR and field data and four machine-learning (ML) algorithms. Topographic, vegetation, hydrologic indices were used as variables subsequently calibrated and validated using field samples.

A complete overview of the methodological flowchart can be found in Fig. 3. The method used is discussed in detail in the following subsections comprising data pre-processing, classification, training data, and statistical models used for prediction and validation. In this study, we adapted the methodology suggested by Mwita et al.^38^ for small inland wetlands of East Africa; and revised it according to the one proposed by Adeli et al.^39^, LaRocque et al.^30^, and Garba et al.^32^. We combined Sentinel-1 and -2 and ALOS-2 PALSAR data; four statistical classifiers were used. The first step was downloading satellite images. These images were obtained from the ESA and JAXA websites (https://www.eorc.jaxa.jp/ALOS/en/index_e.htm). Once obtained, the processing follows as the second step. It consisted of optical image extraction, correction, and band merge. The corrected bands were clipped to the study area (South-Kivu shapefile obtained from RGC: http://rgc.cd/), and indices were calculated. The indices calculation is presented in Supplementary material S3. The indices formulas were extracted from (https://custom-scripts.sentinel-hub.com/custom-scripts/sentinel-2/indexdb/). In total, 27 indices were used.

#### Distribution data

The term "wetland" used in this study is based on the definition suggested by Steinbach et al.^48^, Chuma et al.^5^, and Amler et al.^7^, and which is currently used in eastern Africa and adapted from the Ramsar definition^49^: 'wetlands are areas of marsh, swamp, inundated valleys, peatland or water, whether natural or artificial, permanent or temporary, with static or flowing water, the depth of which at low tide does not exceed 6 m'. Conceptually, the "potential distribution, existing wetlands" method has served as the foundation for this study. It concentrated on prospective wetlands, representing wetlands' size at the most significant level before human interference. In other words, regions where water-related ecosystems are most likely to develop, are highlighted through prospective wetland mapping. Archive points and polygon files constitute the presence data, which were collected by scientists, environmental local non-government organizations (NGOs), and mostly during our field works from September 2020 to August 2022. Other visual fieldworks of well-known sites were made using drone images and delineated in ArcGIS and Google Earth following the digitalization process. In total, 550 samples shapefiles were considered (Supplementary data S4). For inaccessible wetlands, the location's geographic coordinates were taken from the edge and placed in the center once the image was obtained. More points were taken into consideration for the wetlands that had a large area.

The total sample (shapefiles) was split into two datasets; 70% of plots (385 shapefiles) were used as training samples, and the remaining (30%: 165) as validation samples. Those points were considered "presence samples". The "Absence" data comprised 2300 shapefiles extracted from the RGC comprising cities and main towns, schools, hospitals, villages, airports, farms, tree plantations, woodland, etc. These ground-truth surveys were conducted during the same period as the image acquisition.

#### Data on environmental variables

Since wetlands are characterized by specific topography, vegetation, and hydrology, topographic, vegetation, and hydrological indices were used during the mapping and delineation process. Topographic variables were derived from the digital elevation model (DEM) from ALOS-PALSAR (12.5 m resolution). This comprises the elevation (m), the slope (%), land aspect, curvature, Topographic Witness index (TWI), also known as the compound topographic index (CTI), estimated following the formula: TWI = (αtan*b*) where α is the local upslope area draining through a certain point per unit contour length and b: the local slope in radians. TWI is related to soil moisture influencing rapid runoff and flash floods^50^. It was calculated in ArcGIS 10.7 Esrti-TM. The ALOSPALSAR DEM was first projected, and the flow direction and flow accumulation were calculated. TWI was calculated as the ratio between the Flow accumulation and the slope. For Sentinel-1, the horizontal transmit and horizontal receive polarization (HH), horizontal transmit and vertical polarization (HV), and vertical transmit and vertical receive polarization (VV); the ratio γ =|*HH*|^2^|*VH*|^2^ and η =|*VV*|^2^|*VH*|^2^ were also calculated and integrated. For ALOS-2 PALSAR data, three features were integrated: HH, HV, and HH/HV (ρ =|*HH*|^2^|*VV*|^2^). They were corrected using Freeman–Durden (following surface scattering and double-bounce scattering), Cloude–Pottier (polarimetric decompositions and the compact polarimetric simulations) comprising alpha, beta, gamma, and lambda, multi-polarizations, dual polarizations, and polarimetric decompositions^51^. The SAR data preprocessing comprised speckle reduction, terrain correction, and geocoding following steps developed by Veci^52^, Foumelis et al.^53^, and Braun^54^. Since the province typically has two seasons, one dry season lasting from May to August and another dry season for the remaining months, two images were used, comprised of those taken in July (at the height of the dry season) and one in November (during the high rainy period).

This study considered hydrogeomorphic (HGM) wetland types: riverine, depression, slope, flat, and lacustrine fringe. Wetlands were mapped by modeling groundwater using environmental variables. The standard algorithms implemented steps in S1 Toolbox software include applying orbit files, removing low-intensity noise and invalid data on scene edges, removing thermal noise, radiometric calibration, and orthorectification^51,54^. The correction was made in the Sentinel Application Platform (SNAP) for optical data, starting with Rayleigh correction (computing the bottom of Rayleigh reflectance bands). The image resolution (in m) was resampled at 10 m. The sea level pressure and ozone were maintained at 1013.25 (in hPa) and 300 (in DU). Finally, radiance-to-reflectance conversion was used before indices calculation^55^. The dataset of exploratory variables included 27 environmental variables divided into 7 topographic and Hydrogeomorphology (HGM) variables, 8 SAR, and 12 vegetation indices. The description, formula, and source of these indices are presented in the Supplementary material S3. The radar backscattering was made following Sigma-nought (σo). It is referred to as the radar backscatter per unit area (m^2^/m^2^), expressed in decibels (dB). The standard formula used to calculate σo: σo = 10 * log10(DN2) + K, where DN is the image pixel digital number measured in the SAR amplitude image, and K is a calibration factor that varies depending on the SAR sensor and processor system used. As we use both ALOSPALSAR and Sentinel-1, the factor was fixed to − 83.0 dB and 0 dB, respectively.

**Figure 4:**
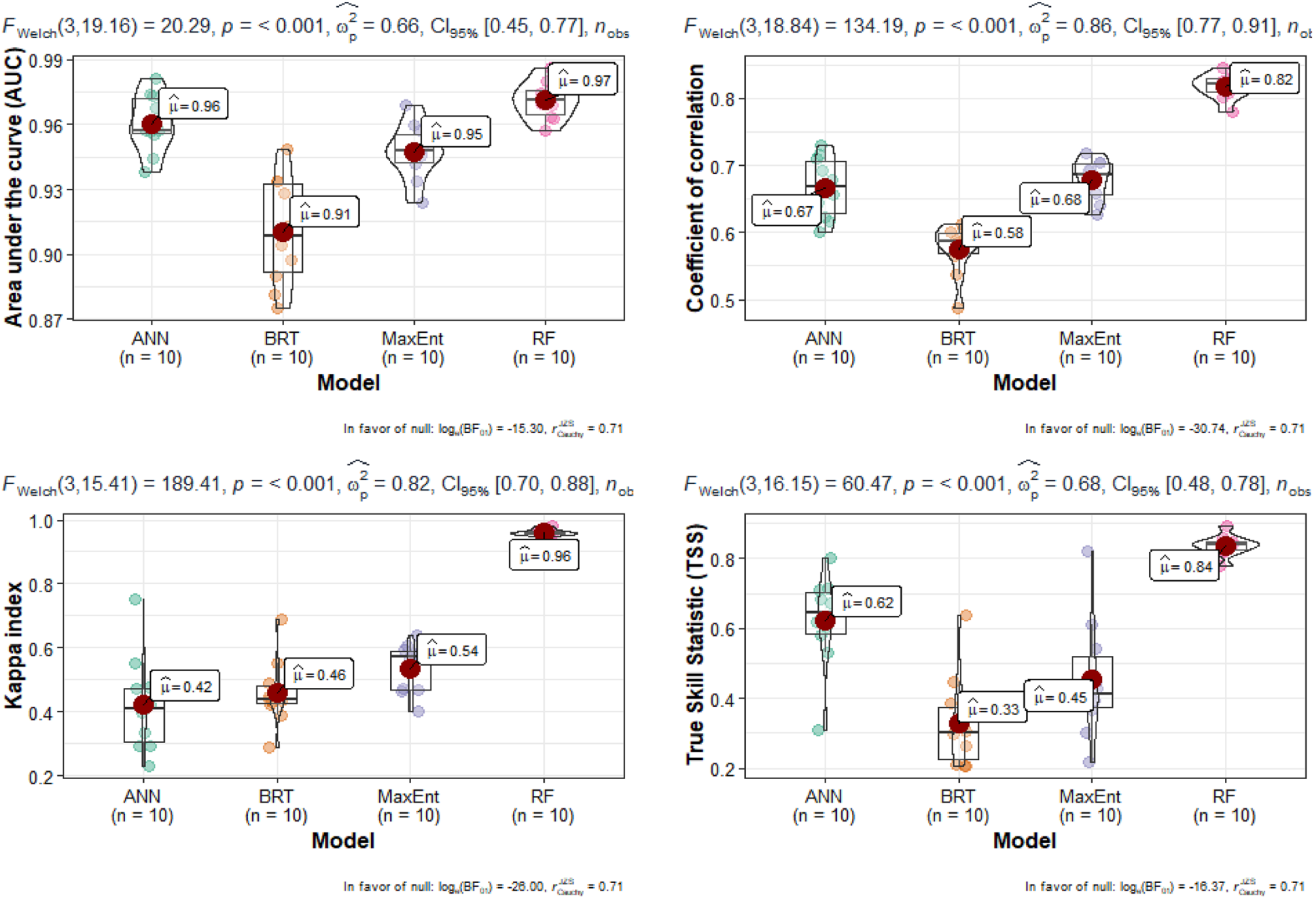
Model performances using AUC, Kappa and TSS, correlation coefficients. The four models were compared with the F-test of Welch and DeLong test. For each model 10 experiments were executed and helped for statistical comparison.

Other ancillary information was used, including GPS points, on-site images obtained from drone missions (Fig. 4), field notes on dominant vegetation, accessibility from roads, villages, or markets, and land use. The GPS points were inserted into ArcMap and google earth, and then the boundary delineation was conducted using high very resolution images. The Universal Transverse Mercator (UTM), zone 35S, was used for projection, while the Geographic Coordinate System (latitude–longitude) WGS1984 was maintained for points. Three non-wetland classes: deep water, urban, and upland, were obtained and merged into one class called 'no-wetland'.

### Model selection and construction

Four spatial statistic models were used comprising Artificial Neural Network "ANN"^21^, Boosted Regression Tree "BRT"^56^, Random Forest "RF"^11,56^ and Maximum Entropy 'Maxent'^35^. It is a computing system inspired by the biological neural networks that make up animal brains, known as neural networks (NNs) or neural nets.

(a) ANN is a widely used machine learning (ML) algorithm that can work for big data analysis. The multilayer feed-forward feature is the primary form of neural networks. ANN includes several neurons or nodes that function in parallel to convert the input data into output types. Typically, ANN consists of three-layer types, namely (i) the input, (ii) the hidden, and (iii) the output layers. Depending on the specific application in a network, each layer has some neurons. Each neuron is connected to other neurons in the next consecutive layer by direct links. These links have a weight that represents the strength of an outgoing signal^57^.

(b) The Random Forest (RF) model has been widely used to map this ecosystem^58,59^. According to Rapinel et al.^11^, due to its ability to consider a large number of variables from many sources and low sensitivity to outliers and over-learning, RF models have been more effective for mapping wetlands than other types of models like support vector machines (SVM), maximum likelihood, or decision trees (DT).

It is a non-parametric supervised ML algorithm inside RStudio and R 4.2.1^60^. Because it can manage the significant difference variables and be used to neutralize noisy data, RF has demonstrated its use for classifications with the enormous volumes of data in satellite images. The input variables for the Random Forests were indices generated from the satellite images and sample points (presence and absence). The number of trees used in each Random Forest classification was set at 250, which has been found to be the ideal amount when accuracy and processing speed are considered. To reduce the tree depth, the minimum node size was set at five. The RF algorithm builds numerous bootstrapped, de-correlated random decision trees to categorize a dataset according to the mode. Indeed, when implementing the RF classifier must specify the number of decision trees and randomly chosen variables for dividing the trees. The RF technique was developed with 250 decision trees utilizing the stratified random sample points after multiple iterations and fine-tuning. An ensemble of 10 models was generated for each combination. The number of training samples was set to 5000 and maintained 10 RF models.

(c) Boosted Regression Tree (BRT): To maximize prediction accuracy while revealing information about pertinent variables and their interactions, BRTs adaptively construct several; basic regression-tree models and merge them into a multi-tree model. This differs from conventional regression methods, which result in a single prediction model. With the added benefits of boosting, which makes it possible to model nonlinear functions and improves robustness to data concerns like outliers, BRTs combine the flexibility of regression trees to accept all data types inside a model, including missing or non-independent data. BRTs are described in depth and detail by Berhane et al.^59^. A BRT model needs two crucial inputs: learning rate and tree complexity. While the latter identifies the number of nodes inside each tree and sets the number of variable interactions that are fitted, the former establishes each tree's contribution to the final model^60^. As each tree contributes less to the overall model with a slower learning rate, more trees are in a BRT model. However, building the model requires more computing effort and observations the more trees there are. Averaging 550 trees per model, we used a learning rate of 0.005 and a tree complexity of 5. In order to reduce the inherent stochasticity in each model because of the subsampling and bagging that go into the construction of each tree, an ensemble of 10 models was created for each combination of inputs and then averaged. Boosted regression trees (BRT) combine the strengths of two algorithms: regression trees (models that relate a response to their predictors by recursive binary splits) and boosting (an adaptive method for combining many simple models to give improved predictive performance). The final BRT model can be understood as an additive regression model in which individual terms are simple trees fitted in a forward, stage-wise fashion.

(d) Maximum of Entropy (Maxent): Currently used for species distribution, Maxent assumes all presence locations of the same type (usually species), and there is little variability in the species' niche preference for locations. It assumes that environmental conditions at extant wetland locations represent the fundamental niche for a particular type of wetland. Maxent cannot adjust for differences in sampling effort type of wetland. In our case, the remotely sensed wetland presence data ensured an unbiased sample, while modeling limitations were collectively addressed using extensive wetland presence coverages to perform validation studies. In this study, we have adapted the methodology developed by Rebelo et al.^61^. We set the number of iterations to 5000, allowing the model enough time to converge. All other Maxent settings were left at their default values. Ten models were also maintained. This technique uses environmental data (here variables) for several "background" sites and known presence locations. The raster files contain variables that were extracted. The result, which was displayed as a probability raster, was reclassified into two classes above-mentioned.

Data were first processed in ArcGIS 10.7 Esri-TM and R 4.2.1 to run the models. The R packages "raster", "rgdal", "virtual species", RStoolbox", "xlsx", and "data.table" help to process the images and the training samples. These packages also helped with data stacking, data frame creation, and raster processing to the same extent, resolution, and projection system. All the data were brought to WGS84/UTM zone 35S. To run the models, the package "MASS", "gbm", for BRT, "dismo", "SDMtune", "NMeval", "virtualspecies", "maxnet" for Maxent, "neuralnet" and "caret" for ANN, "randomForest" and "caret" for RF. For the result presentation, "ggplot2" and "gpairs" helped design graphs. The output files are probability images (ranging from 0 to 1) that were split into two classes, "wetland" and "non-wetland"^1,35^.

### Post classification

The post-classification stage of this study aimed to refine the wetland inventory by removing erroneously classified pixels. Indeed, before conversion, post-classification was made. Three spatial analyses were made; we started first with the "majority filter tool" followed by the "boundary clean tool". The wetland shapefiles were smoothed using the boundary between areas by expanding and shrinking. The small clusters were processed using the "region group tool"^62^. The "polynomial approximation with exponential kernel smoothing algorithm" was finally used to smooth wetland shapefiles.

### Accuracy assessment

To determine the level of accuracy of the small inland wetland map, it is necessary to validate the predicted wetted landscape using optical and SAR imageries. The second dataset (30%: 165) of wetlands was used for validation. These wetlands were physically surveyed to explore their nature and water seasonal availability to validate the predicted wetland areas. 300 sites were collected from Google Earth Pro images and selected from a field survey using GPS. They were used to assess the overall and local accuracy. The accuracy was assessed using the Area under the curve (AUC), Kappa coefficient, correlation, and True Skill Statistics (TSS). First, we started assessing the "Sensitivity" and "Specificity". In fact, due to their complementarity and the fact that they comprise the core of the Receiver Operator Characteristic "ROC" curve, Specificity and sensitivity are two fundamental metrics in classification^63,64^. Sensitivity and Specificity in this study relate to the accuracy of the "wetland" and "non-wetland" classes producer, respectively. Both concepts have their roots in the literature on ecological presence-absence distribution models, such as species and habitat distribution models^65^, which are comparable to the probability of wetland-occurrence models developed. Here, sensitivity is a term used to define the percentage of presences (wetlands) identified; it measures the degree of omission errors. Specificity is the fraction of accurately anticipated absences (i.e., non-wetland) that occurred. The formula (i) and (ii) are used to ass the two parameters and combined in the confusion matrix as presented below. The two parameters are True Positive Rate (TPR) and False Positive Rate (FPR). Sensitivity is generally used to describe the proportion of observed presences (i.e., wetlands) that are correctly predicted as such and is a measure of the level of omission errors. Specificity, on the other hand, is a measure of errors of commission, representing the proportion of correctly predicted absences (i.e., non-wetland).

Sensitivity = $\frac{\alpha }{\alpha + \delta }$ (i) and Specificity = $\frac{\beta }{\beta + \gamma }$ (ii), with n = α + β + γ + δ. α (TP) is the true presence, β (TN): true negative, γ (FP): the false positive and δ (FN): the false negative.

*AUC:* is a comprehensive assessment of the model's overall performance. It provides a conceivable categorization threshold and is interpreted differently; one is the probability that the model classifies a positive example more carefully than a negative one. AUC is a robust and commonly used measure of predictive model performance. The AUC ranges from 0 to 1. An entirely false prediction model will have an AUC of 0, whereas an accurate prediction model will have an AUC approaching^1,63,64^. The value 1 specifies that the diagnostic test is perfect, 0.5 stands for a worthless test, and 0 signifies the result is entirely wrong. According to Kanti et al.^65^, the AUC value of 0.90–1.00 indicates excellent, 0.80–0.90 means good, 0.70–0.80 means fair, and 0.60–0.70 means poor accuracy level.

*Correlation coefficient (r*^**2**^**)**: The correlation coefficient (r^2^) is a standardized measure of the predictive accuracy of a model. The formula (iii) was used to assess the r^2^.

$${\mathbf{r}} = \frac{{\sum {\left( {{\text{Y}}_{{{\text{sim}}}} - \overline{{{\text{Y}}_{{{\text{sim}}}} }} } \right)\left( {{\text{Y}}_{{{\text{obs}}}} - \overline{{{\text{Y}}_{{{\text{obs}}}} }} } \right)} }}{{\sqrt {\sum {\left( {{\text{Y}}_{{{\text{sim}}}} - \overline{{{\text{Y}}_{{{\text{sim}}}} }} } \right)^{2} } } \sqrt {\sum {\left( {{\text{Y}}_{{{\text{obs}}}} - \overline{{{\text{Y}}_{{{\text{obs}}}} }} } \right)^{2} } } }}\quad\hfill \left( {{\text{iii}}} \right)$$

Y_obs_ is the wetland, and Y_sim_ is the value from the model. Ȳ_obs_, Ȳ_sim_ are the average values of Y_obs_ and Y_sim_. As for AUC, r^2^ varies from 0 to 1. 0.90–1.00 indicates excellent, 0.80–0.90 means good, 0.70–0.80 means fair, and 0.60–0.70 means poor accuracy level^66^.

*Kappa statistic:* The Kappa coefficient is more valuable than the overall accuracy as it indicates how the classification rate compares to the likelihood of correctly classifying pixels by chance. The formula (iv) was used to assess the Kappa coefficient.

$${\text{Kappa}} = \frac{{2 \times \left( {{\text{TP}} \times {\text{TN}} - {\text{FN}} \times {\text{FP}}} \right)}}{{\left( {{\text{TP}} + {\text{FP}}} \right) \times \left( {{\text{FP}} + {\text{TN}}} \right) + \left( {{\text{TP}} + {\text{FN}}} \right) \times \left( {{\text{FN}} + {\text{TN}}} \right)}}\hfill\quad {\text{(iv)}}$$

*True Statistic Skill (TSS):* The TSS, often called the Hanssen-Kuipers discriminant, compares the number of correctly classified samples to that of a hypothetically perfect classification while removing those correctly classified samples that could be attributed to a chance agreement. Equation (v) below is used to calculate the TSS, which provides a more objective measurement of classification accuracy comparable to the Kappa coefficient often employed in the literature on remote sensing.

$${\text{TSS}} = {\text{Sensitivity}} + {\text{Specificity}}{-}1\quad {\text{or}}\quad TSS = (\alpha \times \beta ) - (\beta \times \gamma )(\alpha + \gamma ) \times (\beta + \delta )\hfill\quad {\text{(v)}}$$

## Results

### Contribution of environmental variables

The study employed a rigorous variable selection process to identify the most influential variables for the modeling procedure. Of all the variables integrated into the models, 27 were integrated into the modeling process. The process used for variable selection comprises the Pearson correlation (r) calculation followed by the distance calculation (D = 1 − Pearson’s r). A two-by-two variables comparison, with D < 0.3 (r > 0.7), was used for variable selection. The tree plot that exhibited strong correlations was generated and provided in the additional data. If two are highly correlated (r > 0.7), only one variable was selected to avoid data multi-collinearity (Supplementary material S5). From 27 indices, only 15 were selected. Indeed, DGM, BBLUE, GNDVI, NDWI, and RVI were highly correlated, and only NDWI was selected. WET, GVMI, and NBR were also correlated, and WET was selected. NDVI was selected among MSAVI 2, TVI, SAVI, and OSAVI. Explanatory variables (indices contribution) values vary from one model to another. Two SAR indices were integrated to assess the “permanent” and “seasonal” effects. The results indicate that for all the wetland and non-wetland classes, the ratio ρ and η for both seasons performed better than the other two Sentinel 1 variables (ratio γ, HH, VV, or VH). Table 1 presents the contribution of each explanatory variable for the four models.

**Table 1:** Variable importance for both SAR and Optical indices delivered from Sentinel and ALOS-PALSAR.

Types	Model	WET	η	ρ	MDNWI	GOSSAN	EVI	TWI	TRI	SPI	SLOPE	nNDVI	ASPECT	CURVATURE	DEM	NDWI
With SAR	ANN	4	10	8	21	15	7	14	< 1	< 1	9	< 1	3	5	9	< 1
	BRT	4	11	12	23	18	4	13	< 1	< 1	9	< 1	3	5	8	< 1
	MaxEnt	3	19	9	20	28	2	15	< 1	< 1	4	< 1	1	1	5	< 1
	RF	3	12	10	18	11	4	10	< 1	< 1	14	< 1	6	8	11	< 1
Without SAR	ANN	< 1			< 1	< 1	< 1	7	3	5	52	6	9	3	7	5
	BRT	< 1			< 1	< 1	< 1	5	3	4	53	8	8	3	8	5
	MaxEnt	< 1			< 1	< 1	< 1	< 1	1	2	71	3	13	2	6	1
	RF	< 1			< 1	< 1	3	17	6	9	30	9	6	4	6	7

### Evaluation of model prediction results

#### Model prediction

**Figure 5:**
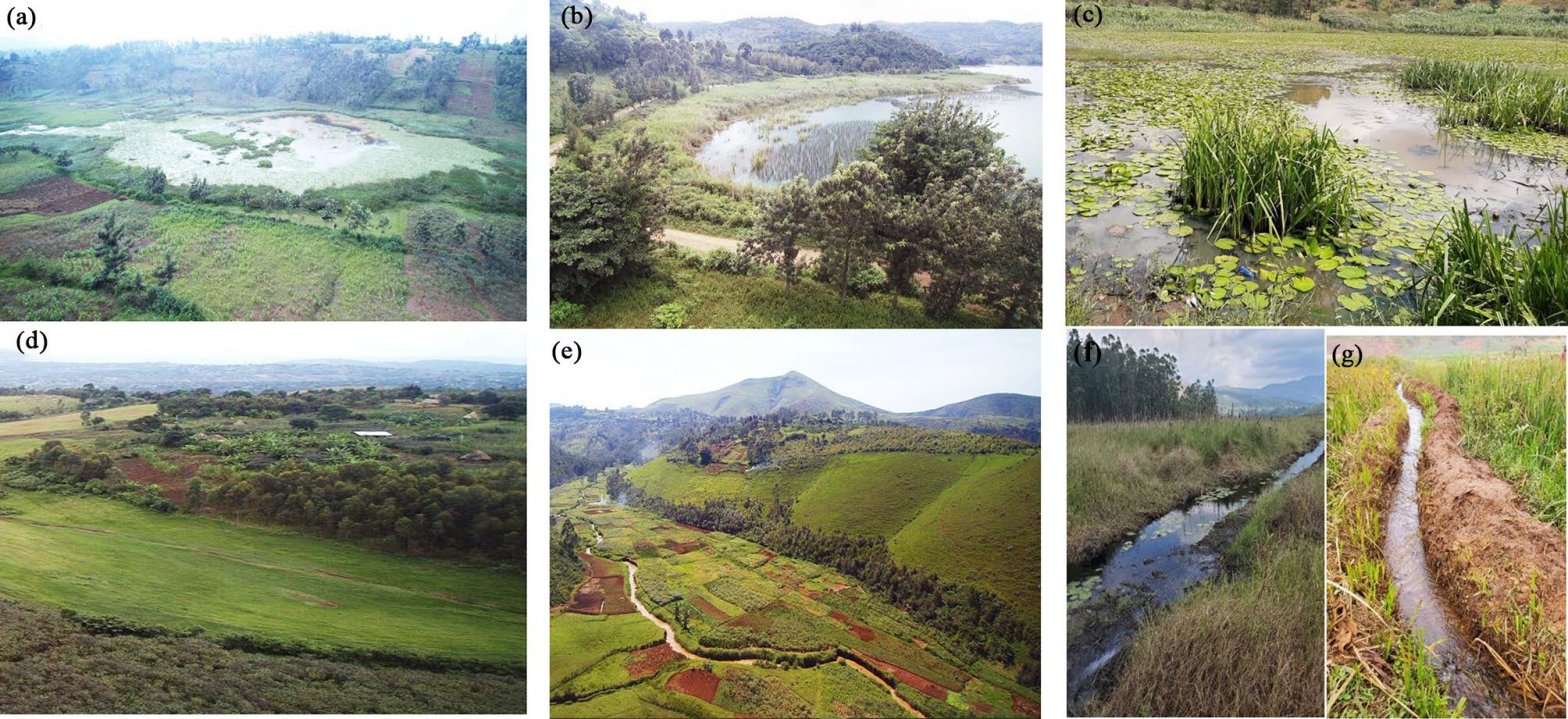
Images obtained from fieldwork when identifying and delineation wetlands in South-Kivu province, here: (**a**) swamp in Kabare, (**b**) papyrus wetland along the Lake Kivu in Kalehe and Kabare, (**c**) marshland in Kabare, (**d**) and (**e**) inundated valley in Walungu, (**f**) peatland in the Nakananda wetland in Walungu, rice valley in Ruzizi plain.

To evaluate the performance of the four models, we started with the accuracy assessment results presented in Fig. 5. First, using a model without SAR data. With 10 experiments for each model, statistical analyses were used for comparison. The AUC varies significantly from one model to another (IC_95_ [0.45–0.77]). More variability was observed in the BRT, ANN, and MaxEnt than in the RF models. The decreasing order of the average AUC values is 0.97, 0.96, 0.95, and 0.91 for RF, ANN, MaxEnt, and BRT, respectively. Thus, even if all the models presented high AUC, the best accuracies were achieved for the experiment from RF. For the coefficient of correlation, the same trends were observed with 0.82, 0.67, 0.68, and 0.58 for RF, ANN, MaxEnt, and BRT, respectively. RF and MaxEnt had acceptable values. Only RF (0.96) presented a high value for the Kappa coefficient compared to all the other models. The same trend was observed for TSS (0.82). Considering the TSS as the final model assessment, it is clear that RF (0.84) has a higher and acceptable value than others. Other models, such as ANN and MaxEnt, presented high variabilities in the experiments. TSS values vary from acceptable to unacceptable values (0.28–0.81). The RF model consistently presented significant values followed by ANN, MaxEnt, and BRT for all model evaluation parameters. Even though all the models generally showed high values for AUC and TSS, they presented very high variabilities (see the height of the boxplot) except for the RF.

Table 1 presents the contribution of explanatory variables. As above-mentioned, from the 27 variables integrated into the models, only 15 were significantly used, and others were omitted to avoid multi-collinearity. 11 variables contributed to RF and ANN models. In contrast, 10 and 9 were integrated for BRT and MaxEnt models. Other variables had minimal contribution (with contribution < 1). Five factors have highly contributed: terrain slope, TWI, GOSSAN, MNDWI, ρ, and η. Topographic indices highly come in the first position. For ANN, ~ 78% of contribution comes from topographic indices, mainly slope (52%), elevation in m (11%), TWI (7%), and Curvature (8%); while for BRT, topography contribution reaches 74% with slope (53%), Aspect (8%), elevation (8%), nNDVI (8%) and TWI (5%). For the MaxEnt model, topographic indices reach 92% of contribution and ~ 5% for vegetation. RF combined more parameters including topography: 68% (slope: 33%, TWI: 17%, Aspect: 6%, elevation: 6%), vegetation (NDWI: 7%, NNDVI: 9%, EVI: 3%).

#### Contribution of SAR and optical data

**Figure 6:**
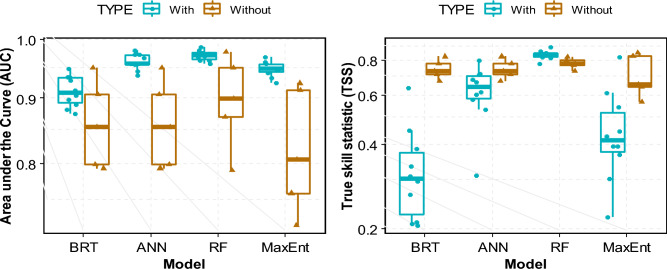
Prediction accuracy of the four models with and without integration of SAR data (here only the AUC and TSS were integrated as accuracy indices).

When integrating SAR data (Fig. 6), the model prediction was significantly improved. RF remains the highly accurate model (AUC: 0.97 and TSS: 0.82), followed by ANN (AUC: 0.85 and TSS: 0.68). When integrating SAR data, the contribution changed significantly; the contribution is shared among different categories of variables. Integrating the SAR data results in a nearly proportional distribution between the indices was mentioned. For RF, the contributing factors are mainly the ratio η: 12%, γ: 10%, NDWI: 18%, GOSSAN: 18%, TWI: 10%, elevation in m (11%), and curvature: 11%) and other variables such as WET (3%), EVI (4%) and Land Aspect (6%). For the second model (ANN), in terms of accuracy, MDNWI (21%), GOSSAN (15%), TWI (14%), and η = 10 have a high contribution. Other variables such as η (8%), elevation (9%), slope (9%), curvature (5%), EVI (7%), WET (4%), and Land aspect (3%) have contributed to the model prediction. The contribution of other explanatory variables for the other models is described in Fig. 2. SAR integration improves the accuracy of models, as presented in Fig. 6.

### Prediction of distribution of inland wetlands

**Figure 7:**
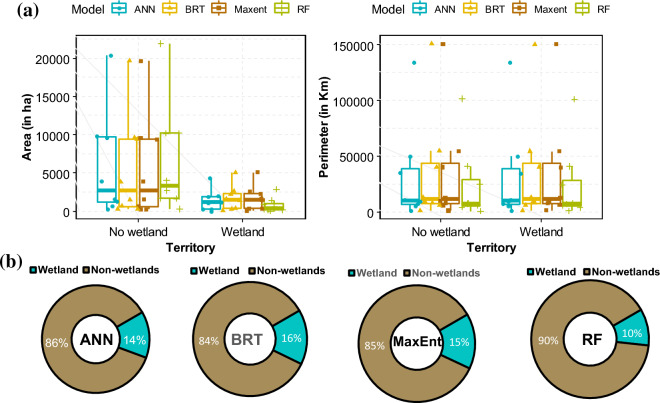
Potential distribution of wetlands in the South-Kivu as predicted by the four models used (the first (**a**) shows the variation per model in term of surface (ha) and Perimeter (Km) after conversion using log2 transformation. The second (**b**) shows proportion of wetland and non-wetland according to each model.

Spatial analysis was employed to characterize the "wetland" and "non-wetland" classes by predicting their area (in hectares: ha) and perimeter (in each territory. The results indicate an average wetland coverage of ~ 13.7% (898,690 ha) in the South-Kivu province, with variations observed across different models (Fig. 7). The ANN model predicted a wetland coverage of ~ 14% (967,820 ha), while the MaxEnt model estimated it to be around 15% (1,036,950 ha). The BRT model resulted in a higher wetland coverage of ~ 16% (1,106,080 ha). In contrast, the RF model predicted a lower wetland area at the provincial scale, with a coverage of 10% (691,300 ha). Considering the higher accuracy of the RF model, one could infer that the first two models, which slightly underestimate the wetland surface area, are more aligned with the actual scenario compared to the other two models.

**Figure 8:**
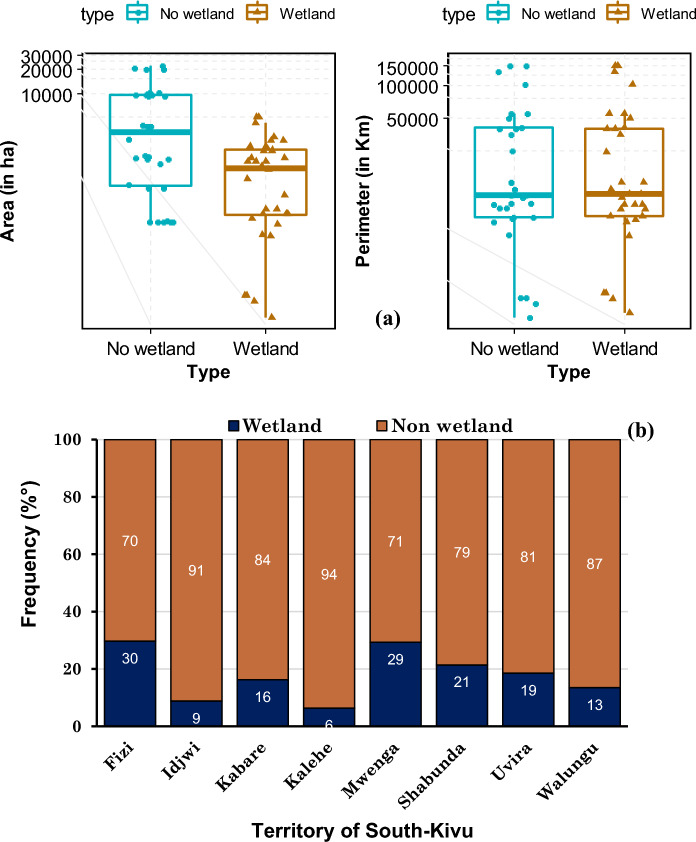
Proportion of wetlands and non-wetlands in the South-Kivu province (**a**) and in the eight territories of South-Kivu province (**b**), (Here we consider only results from RF when we combined optical and SAR data. (**a**) Was obtained after logarithm “log2” conversion of x-axis values for better visualization).

**Figure 9:**
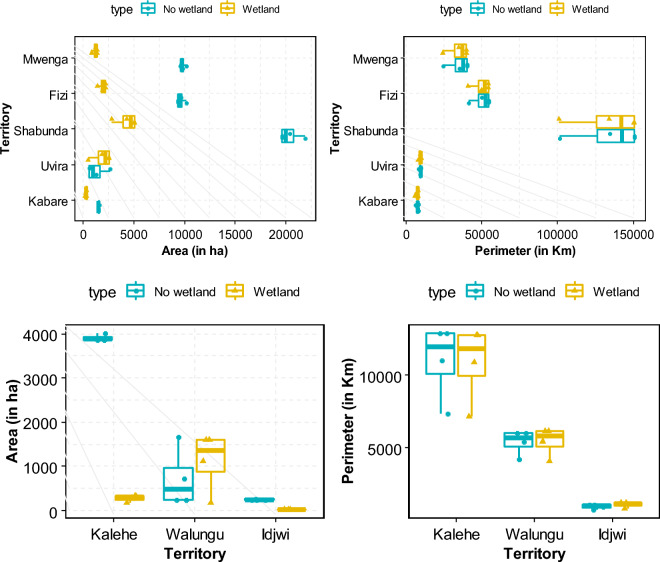
Surface (ha) and perimeter (in km) of the two classes (wetland and non-wetland) after clipped for each territory scale (the eight territories were split here in large and small territory for data representation and visualization.

**Figure 10:**
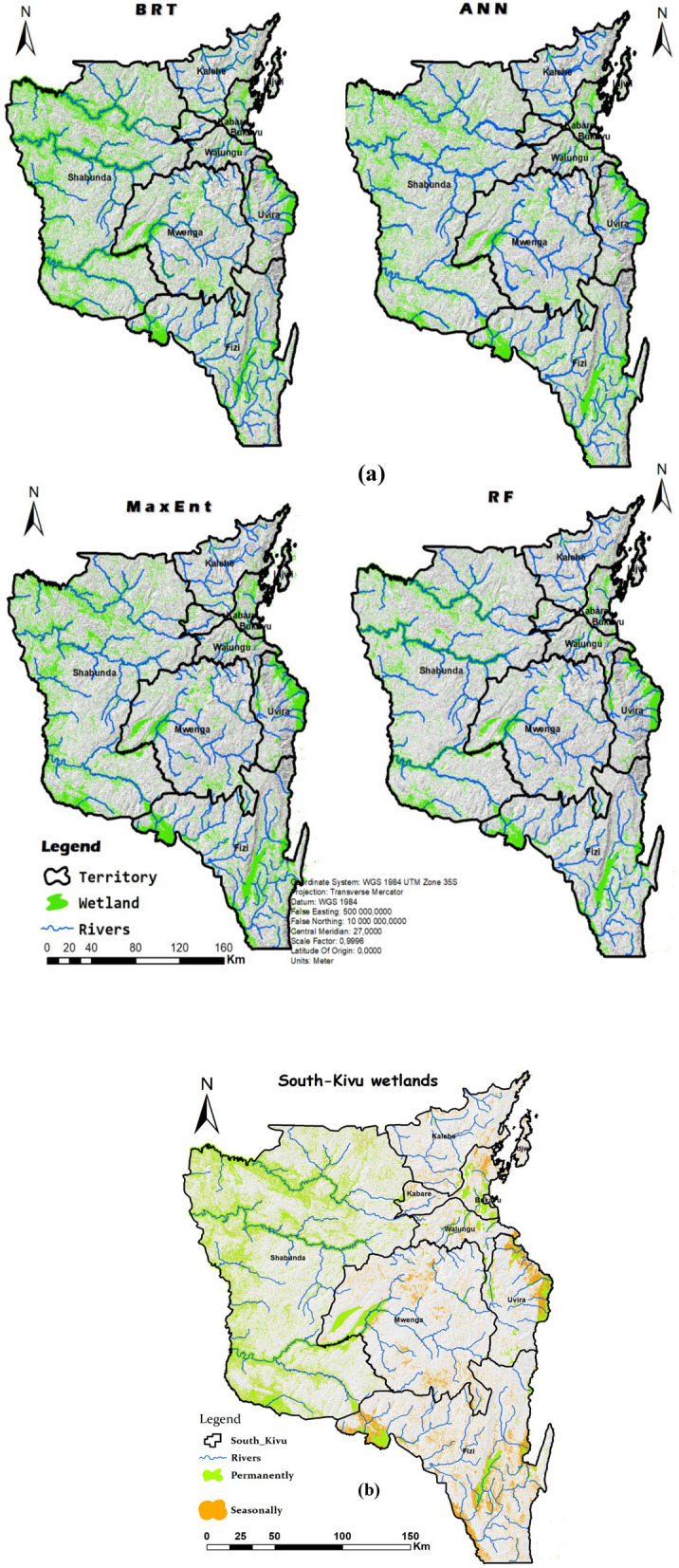
Map of wetland produced in the South-Kivu province, eastern DR Congo using the four models (**a**), and permanently and seasonally flooded wetland map obtained after the integration of SAR data from wet and dry season in RF model (**b**). (Maps created using ArcGIS 10.7 Esri-TM: http://www.esri.com).

The RF model without SAR data predicts that wetland coverage in the South-Kivu province is approximately 10% of the total area. However, when SAR data is integrated, the proportion increases to 13.5%, closer to the merged model's overall mean value. This suggests that roughly 3.5% (241,955 ha) of the province consists of seasonally flooded wetlands. The wetland distribution was then classified and converted to shapefiles, followed by clipping based on each territory. Figure 8b illustrates the calculation of wetland and non-wetland class areas concerning the territory areas. The surface area and perimeter of wetlands vary across different territories. Fizi has the highest wetland surface area at 30% (47,364 ha), followed by Mwenga at 29% (32,398 ha), Shabunda at 21% (52,743 ha), and Uvira at 19% (5981 ha). Conversely, Idjwi has the smallest percentage of wetlands at 9% (252.9 ha), followed by Kalehe at 6% (3075.6 ha), Walungu at 13% (23,400 ha), and Kabare at 16% (31,360 ha). Despite their relatively minor share of the overall area, Shabunda's wetlands cover a larger area than other regions (Fig. 9). The wetland maps generated using the four machine-learning models are presented in Fig. 10a,b.

**Figure 11:**
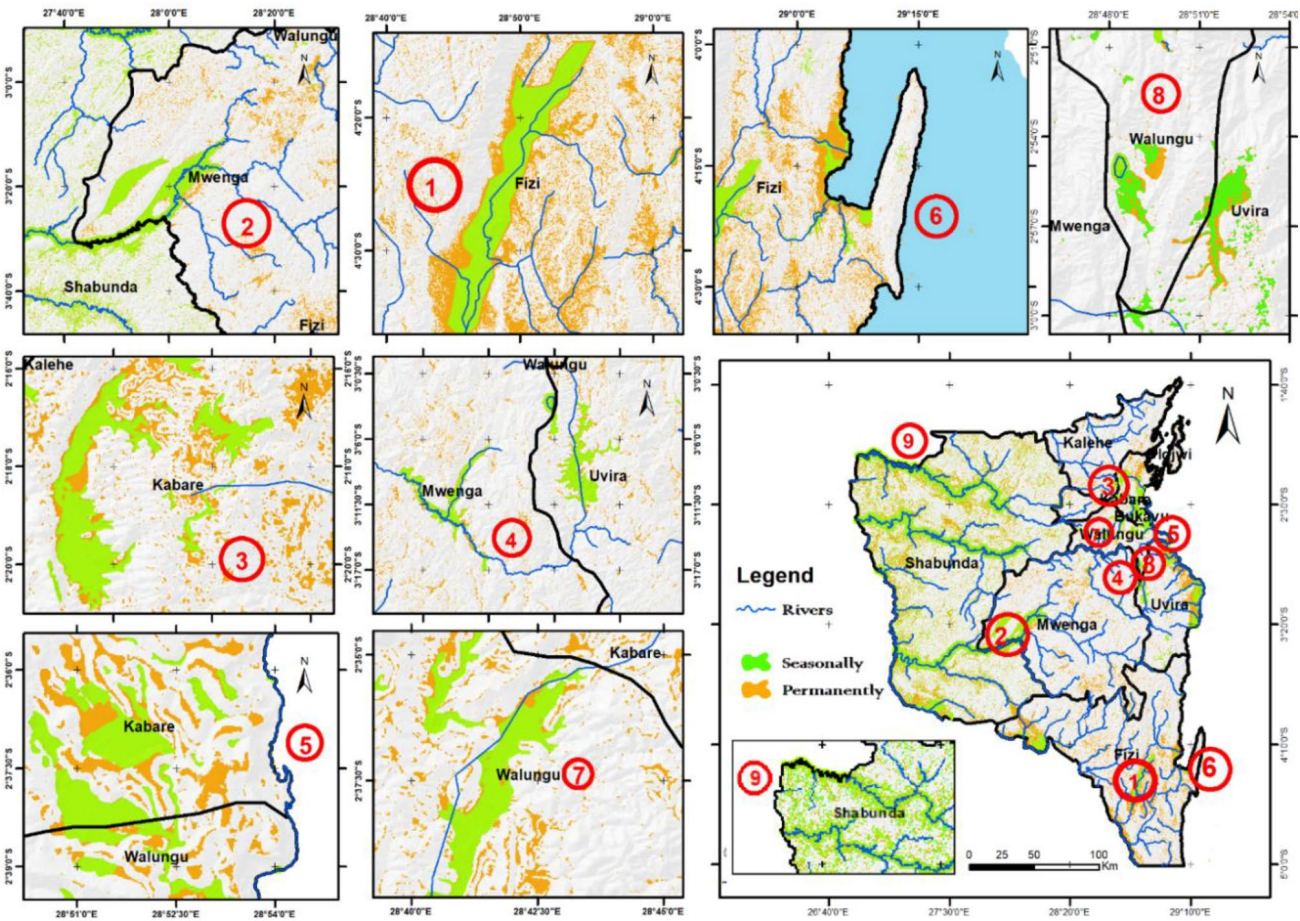
Image showing a zoom in of some wetland complexes that are permanently of periodically flooded in South-Kivu province. (In terms of importance and surface: 1: the Kilombwe and Lubishako rivers’ complex 2: complex around the Nezemere, Kibu, Kalungwe rivers in Mwenga 3: the Musisi, Ngushu, Cishaka complex in PNKB, 4: complex around the Kalungwe river and lake Lubwe in Itombwe, 5: the Hogola, Nyamubanda and 7: Chisheke, Chiherano, and Kachandja, (map created using ArcGIS 10.7 Esri-TM: http://www.esri.com).

Based on these maps, particularly Fig. 10b, the western territories are characterized by permanent wetlands, while the eastern wetlands are predominantly seasonally flooded wetlands. The figure also reveals that seasonally flooded wetlands primarily characterize Uvira, Fizi, and Kabare. In terms of proportions, areas with permanently flooded wetlands, with a persistent layer of water on the soil's surface throughout the year, are more prevalent (~ 68%) than areas with seasonally flooded wetlands. The study area description mentions that the South-Kivu province has protected areas. Overlaying the wetland map with the natural reserves (NRs) in South-Kivu, it is evident that most of these wetlands lie outside of these NRs or protected areas. Figure 10 highlights areas with larger wetland surfaces or multiple small wetland areas grouped, which can be considered "wetland complexes." These areas are present in each territory but are particularly prominent in Fizi, Mwenga, and Shabunda. Examples include the complex near Lake Lubwe and the Kalungwe, Kibu, and Mwana rivers in the NRI (Fig. 11) and some in the KBNP. In Fizi territory, the Kilombwe River complex and Lubishako, situated in the Kabobo Mont (NR of Ngamikka), and another small wetland complex near the Nemba and Mulambala rivers in northern Fizi can be observed. Overlapping the main river shapefile reveals that these wetlands are located along major rivers and coastal lakes, such as the Tanganyika in the case of Fizi. In Uvira territory, the region surrounding the Ruzizi River, particularly the small Ruzizi delta, rich in swamps and peat, and the entire length of the river, are abundant in wetlands. Other wetland complexes in the Mwenga territory include the meanderings of the Elila, Semuliki, Ulindi, Nezemere, Kibu, and Kalungwe rivers: the Kilombwe and Lubishako rivers, the Luama complex, and the Kandja (Fig. 9).

In the northern part of Fizi territory, an additional wetland complex is formed by the Sundja, Mulambala, and Nemba rivers outlets, located along the shoreline of Lake Tanganyika. Uvira territory predominantly features wetlands along the Kiliba, Mulongwe, Sange, and Luberizi rivers. These wetlands have undergone significant transformations and are now mainly utilized as inundated valleys for rice cultivation, and in the case of Kiliba, for sugar cane farming. This entire region is commonly referred to as the Ruzizi valley plain. In Walungu territory, wetlands can be found in Kamanyola (along the Ruzizi River), Kaziba chiefdom, Nyangezi, and Walungu-Ciherano axis. The southern part of the Kabare territory consists of small marshlands within enclosed valleys. The largest wetland complexes in the Kabare territory are still within the Kahuzi-Biega National Park (KBNP) and between the territories of Shabunda and Kalehe. Speaking of Shabunda territory, there are extensive wetland complexes along rivers, including notable ones along the Lugulu, Duma, Ulindi, and Kasema Rivers and along the Mosala River.

**Figure 12:**
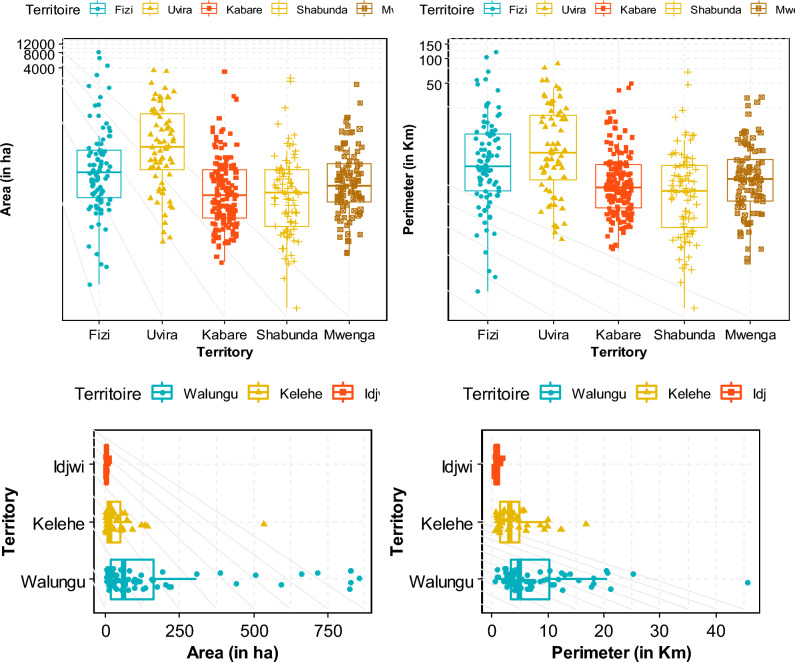
Wetland area in the eight territories of the South-Kivu province, eastern DRC (values were transformed in log2 for better visualization.

The next step involved using high-resolution images to delineate and characterize many wetlands. Each wetland's total surface area and perimeter were determined by calculating the shapefiles obtained from the digitization process and the conversion of the prediction map. The size of wetlands varied greatly, ranging from a few tens to thousands of hectares. In Ijdwi territory, the wetland ranged from 1.3 to 12.3 ha (IC^95^ = [1.4, 5.6], IC_95_: confidence interval of 95%). Kalehe territory exhibited wetlands ranging from 2.4 to 620 ha (IC_95_ = [4.2, 87.8]), while Walungu territory had wetlands varying from 2.5 to 860 ha (IC_95_ = [3.7, 175.2]). Smaller wetlands primarily characterized these territories. The other territories feature wetlands with areas ranging from 5 to 9860 ha and perimeters ranging from 0.8 to 122.6 km. Large wetlands were observed in Fizi, ranging from 1.5 to 8660 ha with an average of 559 ha, IC_95_ = [254, 2600]), and in Uvira, ranging from 1.8 to 2370 ha with an average of 163 ha, IC_95_ = [122.6, 378]). For Shabunda and Mwenga, the average wetland sizes were 5 to 2450 ha (IC_95_ = [58.8, 245.8] with an average of 85.3 ha, and 6.3 to 1781 ha (IC_95_ = [34.3, 132.4] with an average of 71.3 ha respectively. In Kabare, wetland areas varied from 4.1 to 3270 ha with an average of 49 ha (IC_95_ = [120.7, 225.8]) (Fig. 12). Overall, at the provincial scale, the average wetland area and perimeter were ~ 163 ha and ~ 123.6 km, respectively.

Based on fieldwork observations, the wetlands in South-Kivu are characterized by various types, including marshlands, swamps, ponds, peatlands, lakes, river shores, and inundated valleys (Fig. 4). In Kabare and Walungu, specific wetland types such as swamps, marshes, and peatlands were identified, including Cidorho, Irambo, and Chidubo swamps, as well as Nyalugana, Hogola, Nkombo, Kalamba, and Luzinzi marshlands in Walungu. Peatlands were found in Chiherano, Hogola, and Kachandja. These inundated, or floodplain areas are predominantly utilized for rice production (*Oryza sativa* L.).

## Discussion

### Mapping inland wetlands using remote sensing data

The results obtained from our study demonstrate the successful modeling of small inland wetland occurrence in South-Kivu, eastern DRC, by combining optical and SAR indices using machine learning (ML) algorithms. The produced wetland map was reclassified into two classes, ‘wetland’ and ‘non-wetland’, and converted into shapefiles. Among the four ML models tested, the Random Forest (RF) model exhibited high accuracy, with an AUC of 0.97 and a TSS statistic of 0.84, indicating strong discrimination between wetlands and non-wetlands areas. These findings align with the results obtained by Garba et al.^32^ in Nigeria, where a similar approach was employed, and the study area resembles our environment. Eleven out of the 27 indices were used for the RF model, mainly vegetation indices (WET, MDNWI, GOSSAN, and EVI), SAR ratio (η, ρ), and topographic (TWI, slope, land aspect, curvature, and elevation). MNDWI seems to be a good vegetation index for wetland mapping^32^; it contributed up to 21%, 23%, 20%, and 18% for ANN, BRT, MaxEnt, and RF, respectively (Table 1). MNDWI is an index currently used for the enhancement of open water features; the index also diminishes built-up area features that are often correlated with open water in other indices (e.g., when using the G and SWIR bands: pixel values from the green and short-wave infrared band respectively)^67^. In South-Africa, Slagter et al.^68^ used the same approach in combining both Sentinel 1 and 2 and RF and found that 4 explanatory variables (VV, VH; VV/VH, NDVI, and MNDWI) can be used and advised for wetland mapping. However, the problem persisted consistently in wetland areas characterized by the presence of trees. To address the challenge of mapping wetlands in densely forested areas (such as the Shabunda and Mwenga territories), we anticipated that radar sensors operating in high-wavelength L- or P-band with the HH polarization mode would yield more accurate results, as suggested by Slagter et al.^68^. The HH mode enables the observation of double-bounce scattering during floods, essential for mapping wetlands in highly vegetated areas. However, it was anticipated that C-band sensors operating in the VV polarization mode would have limited capabilities for mapping highly vegetated wetlands^68^.

The observation of double-bounce scattering during floods is required for mapping wetlands in densely forested areas (such as Shabunda and Mwenga territories in our case), and this necessitates a certain degree of vegetation penetration of a radar sensor. This is accomplished using high-wavelength L- or P-band sensors with HH mode in highly vegetated wetlands like mangrove or swamp forests. Varied results have also been obtained with C-band and VV mode^68^. It was anticipated in our study that the C-band sensors, operating in η: VV/VH and γ: HH/VH modes, would have only fair capabilities to map highly vegetated wetlands. In our case, η and ρ ratio contributed 10–12% for RF and 8–10% for ANN.

We also anticipated and confirmed that higher-resolution inland wetland mapping with Sentinel and ALOSPALSAR would capture a smaller wetland size than previously documented in regional datasets^38^. Indeed, integrating the SAR improves the accuracy of models. This can be attributed to the improvement in the accuracy of identifying small wetlands vegetation structures and soil water content captured by SAR imagery. Our findings and from Garba et al.^32^ allow us to conclude that the combined use of optical and SAR indices resulted in greater accuracy for small inland wetlands and all wetland classes in general than the use of each one in isolation; such a combination can be advised to produce refined wetland maps. Our final map (Fig. 10) illustrates the spatial distribution of small inland wetlands in South-Kivu province with pixels of 10 m in size. Overall, the classification result shows high accuracy when the RF model is used.

This study's post-classification analysis and digitalization revealed that wetlands account for ~ 13.5% (898,690 ha) of the entire province. These wetlands exhibit a wide range of sizes, varying from a few dozen to thousands of hectares. Most of the wetlands are located in lower altitude territories rather than highlands. However, many small wetland fragments, often-single pixels, were observed, particularly in high-altitude areas. These fragments were reclassified using post-classification methodologies outlined in the methodology section. Regarding the geographic characteristics, it is noteworthy that peats, swamps, and ponds may have developed at very high altitudes. Nzabandora and Roche^69^ suggest that in the territories of Kabare, Kalehe, and Walungu, as well as in extremely high-altitude regions (> 2700 m) in enclosed valleys, these ecosystems can easly form. In these areas, the average temperature can drop below 10 °C at the top of the Kivu's dorsal, while sufficient light, storms, and rainfall maintain an annual precipitation rate of 1600–1700 mm. The presence of significant fault lines in the Lake Kivu sector of the Congo strongly influences the eastern face of the region. The upper courses of these rivers, situated between 2200 and 2300 m.a.s.l, exhibit a senile appearance and give rise to extensive stretches of wetlands, some of which have developed into peatlands. Within these wetlands, native palustrine vegetation, primarily composed of *Cyperus denudatus* and *Cyperus latifolius*, dominates the landscape. These dense vegetation cover in certain areas might explain why traditional vegetation indices, such as the NDVI, SAVI, etc., did not significantly impact the wetland mapping process.

**Figure 13:**
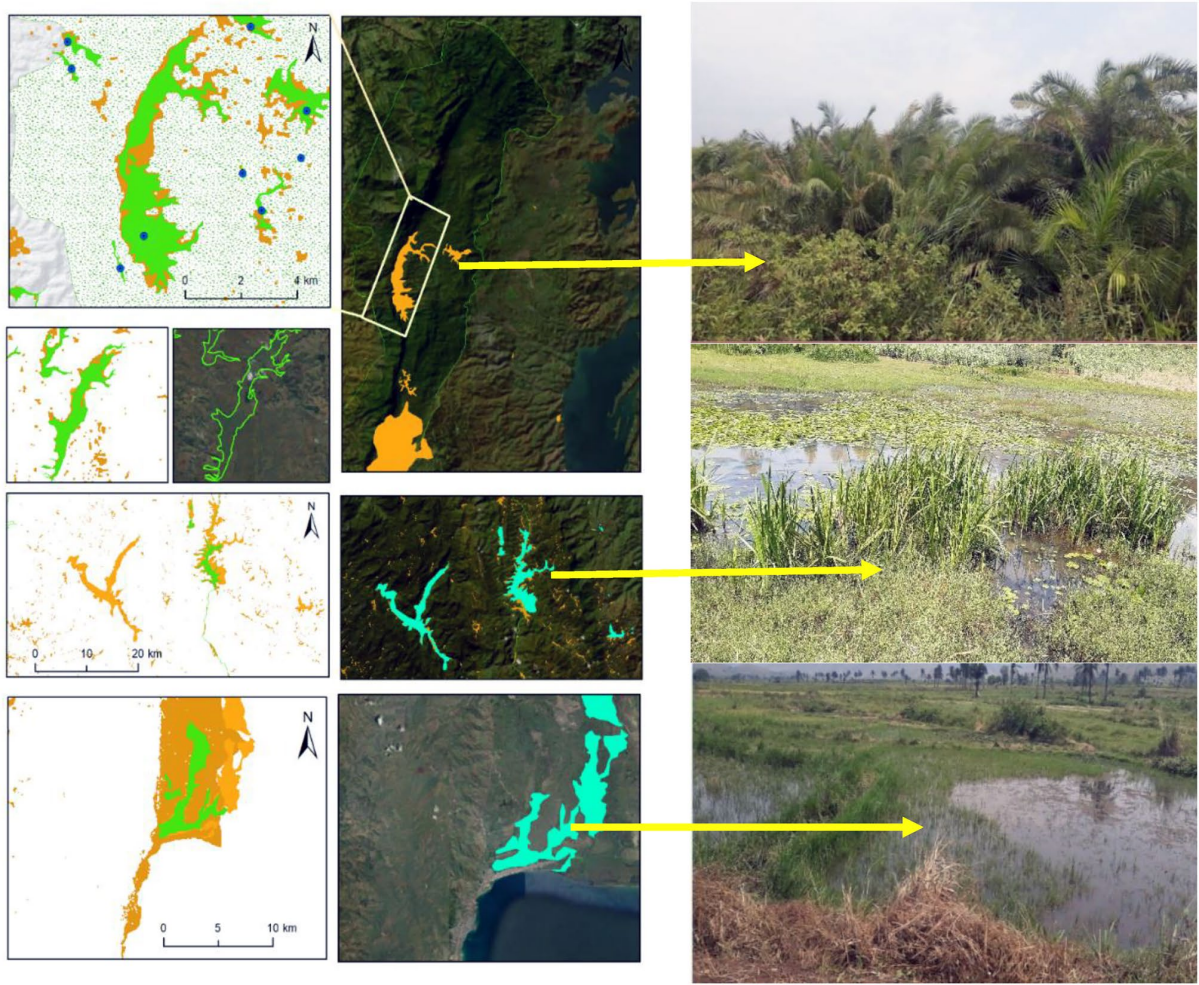
Some wetland complexes located in Kabare, Mwenga and Uvira mainly in the KBNP, NRI and the Ruzizi delta in South-Kivu, Eastern DR Congo (map created using ArcGIS 10.7 Esri-TM: http://www.esri.com).

Nevertheless, gaining a comprehensive understanding of wetland fragmentation in eastern DRC and its impacts on biodiversity, ecosystem services provided to the community, and the role of both larger and smaller inland wetlands in the regional landscape will require further research. The finding from this dataset serves as a valuable starting point for future modeling efforts aimed at enhancing our understanding of these effects. Figures 4, 11 and 13 provide a closer view of some wetland complexes on the wetland map, highlighting their significance despite variations in size and surface area.

The comparison of the produced wetlands map with existing maps in the vicinity revealed interesting insights. It was found that the extent of wetlands in South-Kivu province, as depicted in our study, was more significant compared to some previous studies, such as the one conducted by Kulimushi et al.^46^ and the map available at the Center for International Forestry Research (CIFOR). The CIFOR map was generated at a spatial resolution of 231 m, employing transparent rules on hydrological wetness, satellite-derived soil wetness phenology, and geomorphology. However, the wetlands' extent was underestimated due to its global scale. In contrast, our proposed methodology integrated three biophysical indices that capture essential wetlands characteristics, namely, (i) long-term water supply exceeding atmospheric water demand; (ii) annually and seasonally waterlogged soils; and (iii) a favorable geomorphological position for water provision and retention Gumbricht^70^.

Nonetheless, there is still room for improvement in our methodology. For instance, the inclusion of SAR data captures seasonal variability and enhances the accuracy of wetlands mapping. However, using SAR data is often limited due to their high cost and complex processing requirement^67,71,72^. Nevertheless, recent initiatives such as Sentinel-1 and -2 and ALOSPALSAR have made radar data more accessible for wetland studies. Delineating wetlands through in-situ examination of hydric soil characteristics is time-consuming and expensive, as previously mentioned in our introduction and Lidzhegu et al.^67^. As an alternative, optical remotely sensed images face challenges such as cloud cover and spectral confusion among different land cover categories. To address these issues, incorporating SAR data can penetrate through clouds and provide valuable information. However, providing these data is sometimes possible thanks to Sentinel 1 and 2 and ALOSPALSAR. However, the computational complexities of processing SAR data should be considered^67,71,72^. Regarding the bands used in radar systems, different frequencies are employed for wetland mapping. These include P-band: ~ 69.0 cm (BIOMASS), L-band: ~ 23.5 cm (ALOS-2 PALSAR-2, SAOCOM-1, NISAR-L), S-band: ~ 9.4 cm (NovaSAR, NISAR-S), C-band: ~ 5.6 cm (Sentinel-1, Radarsat-2, RCM), X-band: ~ 3.1 cm (TerraSAR-X, TanDEM-X, COSMO-SkyMed). This study used C- and -L bands for ALOSPALSAR and Sentinel since they are freely available compared to other SAR data^72^.

While our approach did not integrate geomorphological and pedologic variables, we found that topographic indices played a significant role in wetland distribution. Variables such as slope, TWI, Curvature, and other indices like TRI and SPI contributed substantially to the wetland mapping. The compound topographic index (CTI), also referred to as the topographic wetness index (TWI), is particularly relevant as it quantifies steady-state moisture and exhibits strong correlations with various soil characteristics^67,72^. Additionally, Lidzhegu et al. 65 and Ludwig et al.^73^ noted that although numerous spectral indices for water and wet soil detection exist, wetlands can still be easily confused with other land cover types, such as forest and shadows, due to similar spectral profiles. Therefore, our approach incorporated additional auxiliary data in the classification process to mitigate such errors. Previous studies have also highlighted the influence of topographic indices on wetlands mapping. Indeed, Hansen et al.^74^, Rapinel et al.^11^, Guasselli et al.^75^; Berhanu et al.^56^ utilized digital terrain models (DTMs) as explanatory variables to identify estuarine-fringe wetlands. Three topographic variables derived from DTMs were commonly used: (a) the multiscale topographic position index (TPI), (b) the vertical distance to the channel network (VDCN), and (c) the topographic wetness index (TWI), TWI, which characterizes potential soil wetness based on contributing area and local slope, typically ranges from 0 to 30, with higher values indicating a higher probability of wet soil^67^. TWI has proven to be suitable for characterizing riverine wetlands. We integrated two indices, mainly TWI and TPI, which significantly contributed to the wetland mapping process.

The TWI also helps predict soil characteristics, including horizon depth, silt percentage, organic matter content, and phosphorus. The index is used to define biological processes such as yearly net primary production, vegetation patterns, and forest site quality, as well as to explore spatial scale effects on hydrological processes and identify hydrological flow pathways for geochemical modeling^56,75,76^. However, according to Ludwig et al.^73^, even though a variety of spectral indices for water and wet soils detection (hereafter referred to as wetness) is available, wetlands are still easily confused with other upland land cover types such as forests and shadows, since they share similar spectral profiles. In our approach, we have included additional auxiliary data in the classification to minimize these errors significantly.

### Accuracy of ML models

According to the literature review, the four models tested for wetland mapping are commonly used in central and East Africa studies. Among these models, Random Forest ‘RF’ demonstrated higher accuracy than the other three. Only the ANN model comes close to the accuracy of RF. This suggests that RF is the most accurate model for mapping small inland wetlands, and its capabilities in wetland mapping and monitoring have been consistently demonstrated. This conclusion aligns with the findings of Garba et al.^32^ in Nigeria, Barbosa and Maillard^77^ in Brazil, and Slagter et al.^68^ in South-Africa, who also identified RF as a highly accurate model for wetland mapping. Slagter et al.^68^ further discussed the integration of SAR data and found no significant accuracy differences between Sentinel-1 and -2 for mapping surface water dynamics. Our results are similar to Slagter et al.^68^, as we also observed high accuracy when combining Sentinel-1 and -2 data. Whyte et al.^33^ conducted a study in South Africa using both Sentinel-1 and -2, as well as SVM and RF models, with RF demonstrating higher accuracy (83.3% OA, Kappa = 0.72) compared to SVM (79.8% OA, Kappa = 0.68). These accuracies were lower than those achieved with optical data alone but increased when optical and radar data were combined.

Despite using the same RF model, the difference in accuracy between our study and Whyte et al.^33^ could be attributed to several factors. One possibility is the integration of new vegetation indices in our study or the specificity characteristics of our region. It is important to note that complex data patterns can be unique to specific geographies, and a model trained in one geographic landscape may not perform equally well in different geography^33^. In our study, the selection of parameters for the four classifiers (ANN, BRT, Maxent, and RF) allowed for a fairer comparative analysis rather than relying on specific classifier evaluations. This technique has been successfully implemented in other LULC investigations^78–80^. Across all evaluation metrics, RF consistently outperformed ANN, BRT, and Maxent, as demonstrated by the AUC, Kappa, correlation, and TSS values. The statistical tests conducted, such as the DeLong Test, F welch, and Brown-Forsythe tests, further confirmed the significant superiority of RF over the other models (as shown in Fig. 4).

The observed differences in the lowest user accuracies between RF and other models can be attributed to the processing steps of each model. At the same time, both RF and BRT combined decision trees, and BRT started the combination process earlier. The RF help to reduce the variation observed in decision trees: by (i) employing various training samples, (ii) defining sub-ensembles with random characteristics, and (iii) building and combining shallow trees (slightly deep)^59^.

Many potential reasons for the different results of the four models can be mentioned. In fact, in each of these classifiers, there are factors that can affect the classification accuracy. Among these, the image segmentation step, the training sample and feature selection, and the parameters tuning set as advised by Mahdavi et al.^37^. While McNairn et al.^81^ and Adam et al.^33^ mentioned that classification accuracy is not the only thing to be considered on a classifier, the operational monitoring purposes, use friendly, the objective of the study and the type of the study area (here small wetlands), etc. have also to be considered. Nevertheless, these classifiers are among the most famous classification algorithms used for wetland mapping^37^. BRT is a supervised classifier belonging to classification and regression trees (CART), here input data are divided into mutually exclusive groups based on their attributes which is different from RF, a Bayesian statistic assuming that feature vectors of each class are normally distributed. While considered as a CART, RF is considered as an extension of Decision tree (DT). Nevertheless, each of these models has its own strengths and weakness. They differ in term of structure, composition and learning process. The choice should be based on the specific characteristics of the available data, the goals of analysis and resources available. Based on the model performance, the specific of the small wetland context, and the quality of our input data, RF was selected as the best model. Supplementary data 14 presents the strengths and weakness identified for each model used. In summary, RF is an ensemble learning algorithm suitable for various tasks, while Maxent is specifically designed for species distribution modeling, and ANN is a versatile algorithm capable of handling complex patterns in data.

This study presents an affordable and practical technique for accurately delineating small inland wetlands using freely available data at a reasonable spatial resolution. Although Landsat is commonly used for wetland mapping in eastern Africa^82^, we opted for Sentinel-1 and -2 and ALOSPALSAR, despite their lower resolution compared to purchased satellite images from providers such as WorldView, Pleiades, GeoEye, etc. which remain very expensive and almost impossible to obtain at the scale of the entire province. These satellite data sources allowed us to achieve satisfactory accuracy in mapping wetlands.

One advantage of our methodology is that we utilized RStudio packages and freely available scripts, eliminating the need for expensive software. This makes the technique accessible and cost-effective for researchers and practitioners involved in wetland mapping. However, it is essential to note that the RF and ANN required significantly more computation time than other classifiers. In our case, using a desktop computer with Intel(R) core (TM) i7-11800 h CPU with 3.5 GHz processor and 32 Go of RAM, the RF and ANN computations took approximately 22–24 h to complete. This longer processing time could challenge more extensive and long-term studies. More powerful hardware with faster processors and greater RAM capacity could be employed to overcome this issue. Furthermore, the execution time could be reduced by optimizing the algorithm implementation or utilizing more powerful hardware. Including additional variables for monitoring purposes, such as multi-temporal data spanning several years, may also affect processing time. Another potential solution is to explore using the Google Earth Engine (GEE), a cloud-based platform offering extensive geospatial processing capabilities. GEE has the advantage of scalability and efficiency, enabling parallel computing and the integration of various data sources. By leveraging GEE, computational challenges associated with large-scale wetland studies can be addressed, opening opportunities for further development and refinement of the methodology. Despite the computational considerations, our study demonstrates the feasibility of cost-effective wetland mapping using freely available data. As technology continues to advance and more powerful computing resources become available, the efficiency of the process can be improved, facilitating larger-scale studies and supporting ongoing wetland monitoring efforts.

### Study limitation

While this study has contributed significantly to our understanding of the spatial distribution of small inland wetlands in the South-Kivu province, it does have certain limitations that should be acknowledged. One limitation is related to the accessibility of certain territories within the province. Due to the challenges of accessing some areas, fewer training sites were established than in other territories. This discrepancy in training samples could result in variations in precision and accuracy across different territories. Territories closer to urban centers and more accessible, such as Kabare, Walungu, Uvira, and Kalehe, had more training points than Mwenga, Shabunda, and Fizi. This uneven distribution of training samples could introduce bias and affect the accuracy of wetland mapping in different regions. Future research should address this limitation by ensuring a more balanced representation of training samples across all territories.

Another limitation of the methodology used in this study is grouping all wetlands into a single class. While the images in Fig. 4 demonstrate the diversity of wetland types, ranging from lakes and river shore wetlands to peatlands, marshlands, bogs, and swamps, the available training samples for these specific wetland types were limited. For instance, only a small number of peatland samples were identified during fieldwork (~ 12 only). As a result, all these wetland classes were merged to reduce classification errors. Therefore, future research should aim to map and differentiate between these various wetland types to improve wetland maps' accuracy and representational quality.

Additionally, the study lacks lithological, geological, and pedological variables. The available data on these aspects have a relatively low spatial resolution, ranging from 5 to 15 km^83^. Despite the potential relevance of these variables in delineating wetland types, they were not integrated into the analysis due to their limited resolution. However, indices such as TRI and TWI, which are associated with these elements, have been included. It is important to mention that the available soil data have resolutions ranging from 500 m to 5 km. However, we aimed to restrict the resolution of our results to 10 m. Therefore, this decision also justified excluding these data from the integration process.

Furthermore, this study primarily focused on mapping and delineating small inland wetlands without providing a comprehensive characterization of these wetlands, their ecosystem services, and constraints in utilization. To fully understand the extent and functioning of different wetland types and their fragmentation, future research should consider comprehensive assessments incorporating detailed information on wetland characteristics, ecosystem services provided, and the impacts of human disturbances. Such comprehensive wetland mapping and knowledge of their fragmentation patterns are crucial for economic assessments and decision-making by regional and international agencies.

Also, while the study used images from two different seasons to assess the seasonality of inundated areas, there are limitations in capturing the peak of inundations^33^. The periodicity of satellite imagery may not align with the specific timing of peak inundation events. Additionally, the strong interannual variability of African river and water flow regimes poses challenges in accurately detecting inundation extents using publicly available satellite data. Moreover, cloud cover can sometimes hinder the visibility of wetland areas during satellite image acquisition. Addressing these challenges would require the development of robust cloud masking models and potentially exploring alternative data sources or techniques, such as synthetic aperture radar (SAR), to overcome these limitations.

In summary, while this study has advanced our understanding of small inland wetlands in the South-Kivu province, it is essential to acknowledge the limitations related to the distribution of training samples, the grouping of wetlands into a single class, the exclusion of certain environmental variables, and the challenges in capturing peak inundation events and addressing cloud cover^84–86^. Future research should strive to address these limitations and incorporate a more comprehensive approach to wetland mapping and characterization.

## Conclusion

Based on the findings of this study, we can confidently conclude that the mapping of small inland wetlands in the South-Kivu province can be carried out with remarkable precision using a combination of topographic and vegetation indices within a Random Forest (RF) model. By incorporating Synthetic Aperture Radar (SAR) data, we were able to enhance the accuracy and effectively capture the seasonal variations of these wetland areas. Our proposed methodology, which involved carefully selecting a subset of variables and considering new indices, yielded an impressive accuracy rate of approximately 72%. Notably, variables such as η and ρ backscattering ratio, MDNWI, TWI, slope, and elevation played a significant role in achieving these results. Our analysis estimated that ~ 13.5% (equivalent to 898,690 ha) of the South-Kivu province is covered by small inland wetlands. These wetlands exhibit a wide range in size, spanning from a few acres to vast expanses of thousands of hectares. It is important to acknowledge that due to data limitations, we merged different types of wetlands into a single class to avoid introducing bias. Nonetheless, this approach creates exciting opportunities for future research endeavors to delve into the characterization and classification of the diverse wetland types identified during our fieldwork. The significance of wetlands in vital ecological processes such as the water cycle, greenhouse gas exchange, carbon dynamics, and the support of biodiversity cannot be overstated. As such, our study serves as a stepping-stone for further investigations aimed at comprehending the functionality, ecosystem services, and potential risks associated with these wetlands.

Future research should focus on assessing the specific ecosystem services provided by these wetlands, quantifying their contribution to carbon storage, and evaluating their role in supporting and preserving biodiversity. Our findings underscore the importance of understanding and conserving wetland ecosystems, they provide valuable insights for informed decision-making regarding wetland conservation, management, and sustainable land use practices in the South-Kivu province. By understanding these unique ecosystems comprehensively, we can effectively protect their invaluable services, mitigate risks such as floods and pollutants, and harness their potential for climate change mitigation.

### Supplementary Information

Supplementary Information.

## Data Availability

The datasets used and/or analyzed during the current study are available from the corresponding author on reasonable request. The raw data will be provided once the paper is accepted. These data comprise the built scripts in RStudio for data processing, the scripts for the four models, and the shapefiles and raster satellites images used.

## Abbreviations

DRC: Democratic Republic of Congo
SAR: Synthetic Aperture Radar
LULC: Land use and land cover
DEM: Digital elevation model
AUC: Area under the curve
NDVI: Normalized difference vegetation index
NDWI: Normalized difference water index
TWI: Topographic weightiness index
ALOS: Advanced Land Observing Satellite
ASTER: Advanced spaceborne thermal emission and reflection radiometer
DEM: Digital elevation model
GIS: Geographic information system
GLDW: Global lakes and wetlands dataset
HH: Horizontal transmitted and horizontal received (polarization mode)
γ:HH/HV: Horizontal transmitted and two types of receptions horizontal and vertical
NIR: Near infrared
PALSAR: Phased Array L-Band Synthetic Aperture Radar
VV: Vertical transmitted and vertical received (polarization mode)
η:VV/VH: Vertical transmitted and two types of receptions vertical and horizontal

## References

[1] Bwangoy J-RB, Hansen MC, Roy DP, Grandi GD, Justice CO (2010). Wetland mapping in the Congo Basin using optical and radar remotely sensed data and derived topographical indices. Remote Sens. Environ..

[2] Bwangoy J-RB, Hansen MC, Potapov P, Turubanova S, Lumbuenamo RS (2013). Identifying nascent wetland forest conversion in the Democratic Republic of the Congo. Wetl. Ecol. Manag..

[3] Lee H, Yuan T, Jung HC, Beighley E (2015). Mapping wetland water depths over the central Congo Basin using PALSAR ScanSAR, Envisat altimetry, and MODIS VCF data. Remote Sens. Environ..

[4] Dargie GC, Lawson IT, Rayden TJ, Miles L, Mitchard ETA, Page SE, Bocko YE, Ifo SA, Lewis SL (2019). Congo Basin peatlands: Threats and conservation priorities. Mitig. Adapt. Strat. Glob. Change.

[5] Chuma GB, Mondo JM, Sonwa DJ, Karume K, Mushagalusa GN, Schmitz S (2022). Socio-economic determinants of land use and land cover change in South-Kivu wetlands, eastern D.R. Congo: Case study of Hogola and Chisheke wetlands. Environ. Dev..

[6] Balasha AM, Munyahali W, Kulumbu JT, Okwe AN, Fyama JNM, Lenge EK, Tambwe AN (2023). Understanding farmers’ perception of climate change and adaptation practices in the marshlands of South Kivu, Democratic Republic of Congo. Clim. Risk Manag..

[7] Amler E, Schmidt M, Menz G (2015). Definitions and mapping of East African Wetlands: A review. Remote Sens..

[8] Rebelo L-M (2010). Eco-hydrological characterization of inland wetlands in Africa using L-band SAR. IEEE J. Sel. Top. Appl. Earth Observ. Remote Sens..

[9] Wu N, Shi R, Zhuo W, Zhang C, Zhou B, Xia Z, Tao Z, Gao W, Tian B (2021). A classification of tidal flat wetland vegetation combining phenological features with google earth engine. Remote Sens..

[10] Pham HT, Nguyen HQ, Le KP, Tran TP, Ha NT (2023). Automated Mapping of Wetland Ecosystems: A study using Google Earth Engine and machine learning for lotus mapping in Central Vietnam. Water.

[11] Rapinel S, Panhelleux L, Gayet G, Vanacker R, Lemercier B, Laroche B, Chambaud F, Guelmami A, Hubert-Moy L (2023). National wetland mapping using remote-sensing-derived environmental variables, archive field data, and artificial intelligence. Heliyon.

[12] Fluet-Chouinard E, Stocker BD, Zhang Z, Malhotra A, Melton JR, Poulter B, Kaplan JO, Goldewijk KK, Siebert S, Minayeva T, Hugelius G, Joosten H, Barthelmes A, Prigent C, Aires F, Hoyt AM, Davidson N, Finlayson CM, Lehner B, McIntyre PB (2023). Extensive global wetland loss over the past three centuries. Nature.

[13] Lee H, Yuan T, Jung HC, Beighley E (2014). Mapping wetland water depths over the central Congo Basin using PALSAR ScanSAR, envisat altimetry, and MODIS VCF data. Remote Sens. Environ..

[14] Kulawardhana RW, Thenkabail PS, Vithanage J, Biradar C, Islam MA, Gunasinghe S, Alankara R (2007). Evaluation of the wetland mapping methods using Landsat ETM+ and SRTM data. J Spat Hydrol.

[15] García E, Lleellish MA (2012). Mapping bofedales using Landsat satellite images in a Peruvian highandean basin. Revista de Teledeteccion.

[16] Farda, N. M., Danoedoro, P., Hartono, & Harjoko, A. Image mining in remote sensing for coastal wetlands mapping: From pixel based to object based approach. In *2nd International Conference of Indonesian Society for Remote Sensing, ICOIRS 2016* 47(1). 10.1088/1755-1315/47/1/012002 (2016)

[17] Alves GBM, Santos JWMC, Tondato KK, Angeoletto F, Loverde-Oliveira SM (2019). Flood mapping by using land surface water index and water flow characterization in the Pantanal, Brazil. Revista Geografica Venezolana.

[18] Sun C, Li J, Cao L, Liu Y, Jin S, Zhao B (2020). Evaluation of vegetation index-based curve fitting models for accurate classification of salt marsh vegetation using sentinel-2 time-series. Sensors.

[19] López-Tapia S, Ruiz P, Smith M, Matthews J, Zercher B, Sydorenko L, Varia N, Jin Y, Wang M, Dunn JB, Katsaggelos AK (2021). Machine learning with high-resolution aerial imagery and data fusion to improve and automate the detection of wetlands. Int. J. Appl. Earth Observ. Geoinfor..

[20] Islam MM, Ujiie K, Noguchi R, Ahamed T (2022). Flash flood-induced vulnerability and need assessment of wetlands using remote sensing, GIS, and econometric models. Remote Sens. Appl. Soc. Environ..

[21] Saha TK, Pal S, Sarkar R (2021). Prediction of wetland area and depth using linear regression model and artificial neural network based cellular automata. Ecol. Inform..

[22] Hui F, Xu B, Huang H, Yu Q, Gong P (2008). Modelling spatial-temporal change of Poyang Lake using multitemporal Landsat imagery. Int. J. Remote Sens..

[23] Clewley, D., Whitcomb, J., Moghaddam, M., & McDonald, K. Mapping the state and dynamics of boreal wetlands using synthetic aperture radar. In* Remote Sensing of Wetlands: Applications and Advances* 369–398 (CRC Press, 2015). 10.1201/b18210

[24] Martins VS, Kaleita AL, Gelder BK, Nagel GW, Maciel DA (2020). Deep neural network for complex open-water wetland mapping using high-resolution WorldView-3 and airborne LiDAR data. Int. J. Appl. Earth Observ. Geoinfor..

[25] Bradley AV, Andersen R, Marshall C, Sowter A, Large DJ (2022). Identification of typical ecohydrological behaviours using InSAR allows landscape-scale mapping of peatland condition. Earth Surf. Dyn..

[26] Masenyama A, Mutanga O, Dube T, Bangira T, Sibanda M, Mabhaudhi T (2022). A systematic review on the use of remote sensing technologies in quantifying grasslands ecosystem services. GISci. Remote Sens..

[27] Xi Y, Peng S, Ciais P, Chen Y (2021). Future impacts of climate change on inland Ramsar wetlands. Nat. Clim. Change.

[28] Mallick J, Talukdar S, Shahfahad PS, Rahman A (2021). A novel classifier for improving wetland mapping by integrating image fusion techniques and ensemble machine learning classifiers. Ecol. Inform..

[29] Leemhuis C, Amler E, Diekkrüger B, Gabiri G, Näschen K (2016). East African wetland-catchment data base for sustainable wetland management. Proc. Int. Assoc. Hydrol. Sci..

[30] LaRocque A, Phiri C, Leblon B, Pirotti F, Connor K, Hanson A (2020). Wetland mapping with landsat 8 OLI, Sentinel-1, ALOS-1 PALSAR, and LiDAR data in Southern New Brunswick, Canada. Remote Sens..

[31] Larocque, A., Leblon, B., Woodward, R., Mordini, M., Bourgeau-Chavez, L., Landon, A., French, N., McCarty, J., Huntington, T., & Camill, P. Use of Radarsat-2 and ALOS-PALSAR SAR images for wetland mapping in New Brunswick. Joint 2014 *IEEE International Geoscience and Remote Sensing Symposium, IGARSS 2014 and the 35th Canadian Symposium on Remote Sensing, CSRS 2014*, 1226–1229. 10.1109/IGARSS.2014.6946653 (2014)

[32] Garba SI, Ebmeier SK, Bastin JF, Mollicone D, Holden J (2023). Wetland mapping at 10 m resolution reveals fragmentation in southern Nigeria. Wetl. Ecol. Manag..

[33] Whyte A, Ferentinos KP, Petropoulos GP (2018). Environmental Modelling & Software A new synergistic approach for monitoring wetlands using Sentinels-1 and 2 data with object-based machine learning algorithms. Environ. Model. Softw..

[34] Rudiyanto A, Minasny B, Setiawan BI, Saptomo SK, McBratney AB (2018). Open digital mapping as a cost-effective method for mapping peat thickness and assessing the carbon stock of tropical peatlands. Geoderma.

[35] Rebelo AJ, Scheunders P, Esler KJ, Meire P (2017). Detecting, mapping and classifying wetland fragments at a landscape scale. Remote Sens. Appl. Soc. Environ..

[36] Raney PA, Leopold DJ (2018). Fantastic wetlands and where to find them: Modeling rich fen distribution in New York state with Maxent. Wetlands.

[37] Mahdavi S, Salehi B, Granger J, Amani M, Brisco B, Huang W (2018). Remote sensing for wetland classification: A comprehensive review. GISci. Remote Sens..

[38] Mwita E, Menz G, Misana S, Becker M, Kisanga D, Boehme B (2012). Mapping small wetlands of Kenya and Tanzania using remote sensing techniques. Int. J. Appl. Earth Observ. Geoinf..

[39] Adeli S, Salehi B, Mahdianpari M, Quackenbush LJ, Brisco B, Tamiminia H, Shaw S (2020). Wetland monitoring using SAR data: A meta-analysis and comprehensive review. Remote Sens..

[40] Hird JN, DeLancey ER, McDermid GJ, Kariyeva J (2017). Google earth engine, open-access satellite data, and machine learning in support of large-area probabilistic wetland mapping. Remote Sens..

[41] Pashaei M, Kamangir H, Starek MJ, Tissot P (2020). Review and evaluation of deep learning architectures for efficient land cover mapping with UAS hyper-spatial imagery: A case study over a wetland. Remote Sens..

[42] Cox TP (2012). Farming the battlefield: The meanings of war, cattle and soil in South Kivu, Democratic Republic of the Congo. Disasters.

[43] INS. *INS Annuaire statistique 2013–2014. Ministère Du Plan et Révolution de La Modernité* 1–560 (2015).

[44] OCHA. *Statistiques des populations par zones de santé*. 10.18356/fde1527a-fr (2021).

[45] van Engelen, V., Verdoodt, A., Dijkshoorn, K., & Van Ranst, E. *Soil and Terrain Database of Central Africa—DR of Congo, Burundi and Rwanda* (ISRIC and FAO, 2006).

[46] Kulimushi LC, Choudhari P, Mubalama LK, Banswe GT (2021). GIS and remote sensing-based assessment of soil erosion risk using RUSLE model in South-Kivu province, eastern, Democratic Republic of Congo. Geomat. Nat. Hazards Risk.

[47] PICAGEL. *Programme integre de croissance agricole dans la region des grands lacs* (Cadre Fonctionnel (CF), 2016).

[48] Steinbach S, Cornish N, Franke J, Hentze K, Strauch A, Thonfeld F, Zwart SJ, Nelson A (2021). A new conceptual framework for integrating Earth observation in large-scale wetland management in East Africa. Wetlands.

[49] Mandishona E, Knight J (2022). Inland wetlands in Africa: A review of their typologies and ecosystem services. Prog. Phys. Geogr..

[50] Sørensen R, Zinko U, Seibert J (2006). On the calculation of the topographic wetness index: Evaluation of different methods based on field observations. Hydrol. Earth Syst. Sci..

[51] Moreira A, Prats-iraola P, Younis M, Krieger G, Hajnsek I, Papathanassiou KP (2013). A tutorial on synthetic aperture radar. IEEE Geosci. Remote Sens. Mag..

[52] Veci, L. *Orthorectification Tutorial. no. March, 2016*, 187–188 (2015)

[53] Foumelis, M., Blasco, J. M. D., Desnos, Y. L., Engdahl, M., Fernández, D., Veci, L., Lu, J., & Wong, C. ESA SNAP—Stamps integrated processing for Sentinel-1 persistent scatterer interferometry. In *International Geoscience and Remote Sensing Symposium (IGARSS)*, 2018-07(08) 1364–1367. 10.1109/IGARSS.2018.8519545 (2018)

[54] Braun, A. *Sentinel-1 Toolbox TOPS Interferometry Tutorial. January 2020* 1–25 (2021).

[55] Domain, O., Satellite, M., & Processing, I. *Multispectral Satellite Image Processing* 2.1.156. 10.1016/B978-1-78548-102-4.50002-8 (2016).

[56] Berhanu M, Suryabhagavan KV, Korme T (2021). Wetland mapping and evaluating the impacts on hydrology, using geospatial techniques: A case of Geba Watershed, Southwest Ethiopia. Geol. Ecol. Landsc..

[57] Hsu KL, Gupta HV, Sorooshian S (1995). Artificial neural network modeling of the rainfall-runoff process. Water Resour. Res..

[58] Breman L (2001). Random forest. Mach. Learn..

[59] Berhane TM, Lane CR, Wu Q, Autrey BC, Anenkhonov OA, Chepinoga VV, Liu H (2018). Decision-tree, rule-based, and random forest classification of high-resolution multispectral imagery for wetland mapping and inventory. Remote Sens..

[60] R Core Team. *An Introduction to R. Practical Graph Mining with R* (2023).

[61] Rebelo L-M, McCartney MP, Finlayson CM (2010). Wetlands of Sub-Saharan Africa: Distribution and contribution of agriculture to livelihoods. Wetl. Ecol. Manag..

[62] Yamamoto K, Sayama T, Apip A (2021). Impact of climate change on flood inundation in a tropical river basin in Indonesia. Prog. Earth Planet. Sci..

[63] Lobo JM, Jiménez-valverde A, Real R (2008). AUC : a misleading measure of the performance of predictive distribution models. Glob. Ecol. Biogeogr..

[64] Günen MA (2022). Performance comparison of deep learning and machine learning methods in determining wetland water areas using EuroSAT dataset. Environ. Sci. Pollut. Res..

[65] Kanti T, Pal S, Sarkar R (2021). Ecological Informatics Prediction of wetland area and depth using linear regression model and artificial neural network based cellular automata. Ecol. Inform..

[66] Choi C, Kim J, Han H, Han D, Kim HS (2020). Development of water level prediction models using machine learning in wetlands: A case study of Upo Wetland in South Korea. Water.

[67] Lidzhegu Z, Ellery WN, Mantel SK, Hughes DA (2020). Delineating wetland areas from the cut-and-fill method using a Digital Elevation Model (DEM). S. Afr. Geogr. J..

[68] Slagter B, Tsendbazar N-E, Vollrath A, Reiche J (2020). Mapping wetland characteristics using temporally dense Sentinel-1 and Sentinel-2 data: A case study in the St. Lucia wetlands, South Africa. Int. J. Appl. Earth Observ. Geoinf..

[69] Nzabandora CK, Roche E (2015). Six millennia of environment evolving on the western ridge of the Kivu Lake in the Mount Kahuzi area (D.R.Congo) Palynological analysis of the Ngushu sedimentary sequence. Geo-Eco-Trop.

[70] Gumbricht, T. *Mapping global tropical wetlands from earth observing satellite imagery*. Working paper (103), 47. http://www.cifor.org/publications/pdf_files/WPapers/WP103CIFOR.pdf (2012).

[71] Koch M, Schmid T, Reyes M, Gumuzzio J (2012). Evaluating full polarimetric C- and L-band data for mapping wetland conditions in a semi-arid environment in central Spain. IEEE J. Sel. Top. Appl. Earth Observ. Remote Sens..

[72] Lopez-Sanchez, J. M., Ballester-Berman, J. D., Vicente-Guijalba, F., Cloude, S. R., McNairn, H., Shang, J., Skriver, H., Jagdhuber, T., Hajnsek, I., Pottier, E., Marechal, C., Hubert-Moy, L., Corgne, S., Wdowinski, S., Touzi, R., Gosselin, G., Brooks, R., Yamaguchi, Y., & Singh, G. Agriculture and Wetland Applications. In* Remote Sensing and Digital Image Processing* 25, 119–178 (Springer, 2021). 10.1007/978-3-030-56504-6_3

[73] Ludwig C, Walli A, Schleicher C, Weichselbaum J, Riffler M (2019). A highly automated algorithm for wetland detection using multi-temporal optical satellite data. Remote Sens. Environ..

[74] Hansen, M. K., Dennison, P. E., Graves, S. A., & Brown, D. J. Ugandan dambo wetland classification using multispectral and topographic remote sensing data. In *American Society for Photogrammetry and Remote Sensing Annual Conference 2008—Bridging the Horizons: New Frontiers in Geospatial Collaboration*, vol. 1 218–224 (2008).

[75] Guasselli LA, Simioni JPD, Laurent F (2020). Mapping and classification of wetlands using topographic wetness index (twi) from digital elevation models of the gravataí river basin—Rio grande do sul state (Rs). Brazil. Revista Brasileira de Geomorfologia.

[76] Manhas RK, Gautam MK, Kumari D (1970). Two way indicator species analysis (TWINSPAN) of the herbaceous vegetation in an inland wetland ecosystem of Doon valley Himalaya, India. J. Wetl. Ecol..

[77] Barbosa IS, Maillard P (2010). Mapping a wetland complex in the Brazilian savannah using an Ikonos image: Assessing the potential of a new region-based classifier. Can. J. Remote Sens..

[78] Bassa Z, Bob U, Szantoi Z, Ismail R (2016). Land cover and land use mapping of the iSimangaliso Wetland Park, South Africa: Comparison of oblique and orthogonal random forest algorithms. J. Appl. Remote Sens..

[79] Bhatnagar S, Gill L, Regan S, Naughton O, Johnston P, Waldren S, Ghosh B (2020). Mapping vegetation communities inside wetlands using sentinel-2 imagery in Ireland. Int. J. Appl. Earth Observ. Geoinfor..

[80] Munizaga J, García M, Ureta F, Novoa V, Rojas O, Rojas C (2022). Mapping coastal wetlands using satellite imagery and machine learning in a highly urbanized landscape. Sustainability.

[81] Higginbottom TP, Field CD, Symeonakis E, Caporn SJM, Rosenburgh AE, Wright A (2018). High-resolution wetness index mapping: A useful tool for regional scale wetland management. Ecol. Inform..

[82] Guo M, Li J, Sheng C, Xu J, Wu L (2017). A review of wetland remote sensing. Sensors.

[83] Batjes, N. H. *SOTER-based soil parameter estimates for Central Africa—DR of Congo, Burundi and Rwanda (SOTWIScaf, version 1.0)*. 2007/02, iv + 29. http://www.isric.org/isric/Webdocs/Docs/ISRIC_Report_2007_02.pdf (2007).

[84] Zhang T-T, Geng Z, Huang X-R, Gao Y, Wang S-K, Zhang T, Yang G, Zhao F, Zhuang P (2022). Mapping essential habitat of estuarine fishery species with a mechanistic SDM and Landsat data. Ecol. Indic..

[85] Zhang A, Sun G, Ma P, Jia X, Ren J, Huang H, Zhang X (2019). Coastal wetland mapping with Sentinel-2 MSI imagery based on gravitational optimized multilayer perceptron and morphological attribute profiles. Remote Sens..

[86] Gómez-chova L, Amorós-lópez J, Mateo-garcía G, Muñoz-marí J, Camps-valls G, Gómez-chova L, Amorós-lópez J, Mateo-garcía G, Muñoz-marí J, Camps-valls G, Gómez-chova L, Amorós-lópez J, Mateo-garcía G (2017). Cloud masking and removal in remote sensing image time series image time series. J. Appl. Remote Sens..

