# Supplementary material for: Mapping small inland wetlands in the South-Kivu province by integrating optical and SAR data with statistical models for accurate distribution assessment
Source: Sci Rep. 2023 Oct 17;13:17626. doi: 10.1038/s41598-023-43292-7 (PMC10582158; doi:10.1038/s41598-023-43292-7)
Supplement: Supplementary file 1 — Supplementary Information. [file 41598_2023_43292_MOESM1_ESM.docx]

**Supplementary data**

**
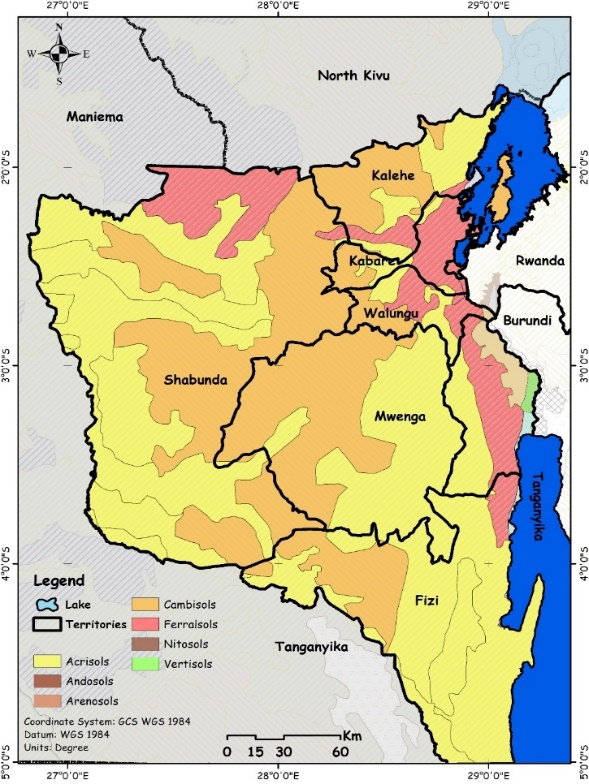
**

**Supplementary material S1.** Climate data (from 2000 to 2019) and soil map of South Kivu province **(map created using ArcGIS 10.7 Esri-TM:** [**http://www.esri.com**](http://www.esri.com)**;** the data were downlord from van Engelen et al., 2006).

**
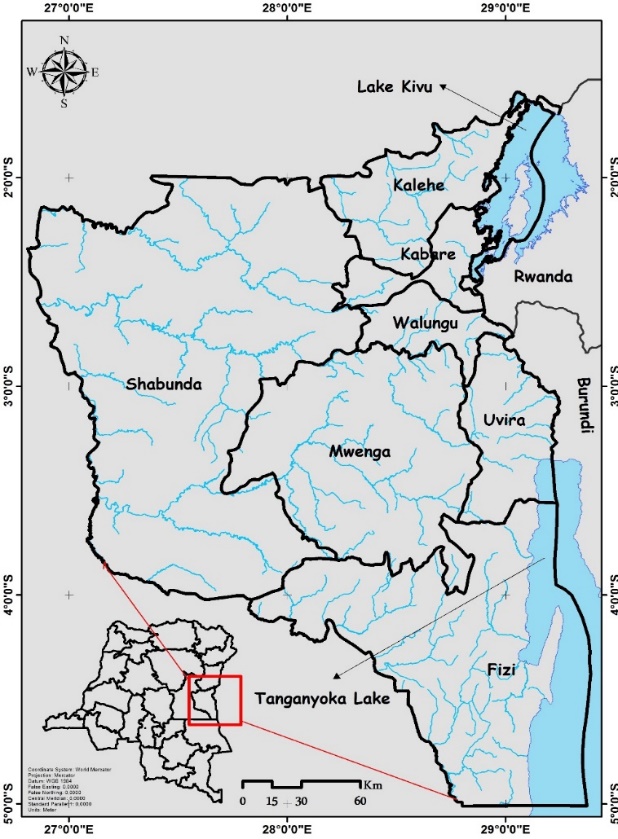
**
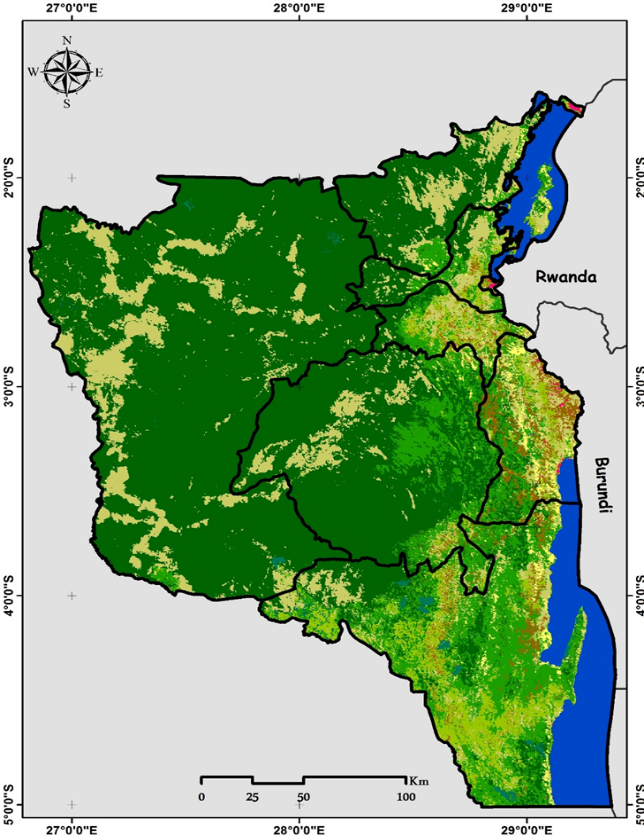


**Supplementary material** **S2:** South Kivu hydrography and land cover and use. **Maps created using ArcGIS 10.7 Esri-TM:** [**http://www.esri.com**](http://www.esri.com)**;**

**Supplementary material** **S3:** Definition and indices formula used in wetland mapping in South-Kivu

| **N°** | **Index** | **Abréviation** | **Formula/tools** |
| --- | --- | --- | --- |
| 1 | LAND ASPECT | ASPECT | Using ArcGIS 10.7 |
| 2 | Band Blue | BBLUE | Bande Bleu (Senyinel 2-A) |
| 3 | CURVATURE | Curvature | Using ArcGIS 10.7 |
| 4 | Elevation | DEM | ALSOSPALSAR |
| 5 | Enhanced Vegetation Index | EVI | $2.5\frac{NIR-RED}{\left( NIR+6RED-7.5BLUE \right)+1}$ |
| 6 | Green Difference Vegetation Index | GDVI | $NIR-G$reen |
| 7 | Green Normalized Difference Vegetation Index | GNDVI | $\frac{NIR-Green}{NIR+Green}$ |
| 8 | GOSSAN | GOSSAN | $\frac{SWIR}{Red}$ |
| 9 | Global Vegetation Moisture Index | GVMI | $\frac{\left( NIR+0.1 \right)-(SWIR+0.02)}{\left( NIR+0.1 \right)+(SWIR+0.02)}$ |
| 10 | Modiﬁed Normalized Difference Water Index | MNDWI | $\frac{Green-SWIR}{Green+SWIR}$ |
| 11 | Modified Soil Adjusted Vegetation Index 2 | MSAVI2 | $\frac{2NIR+1-\sqrt[2]{{(2NIR+1)}^{2}-8(NIR-RED)}}{2}$ |
| 12 | Normalized Burn Ratio | NBR | $\frac{NIR-SWIR}{NIR+SWIR}$ |
| 13 | Normalized Difference Vegetation Index | NDVI | $\frac{\rho NIR - \rho RED}{\rho NIR + \rho RED}$ |
| 14 | Normalized Difference Water Index | NDWI | $\frac{\rho NIR - \rho\mathrm{MIR}}{\rho NIR + \rho\mathrm{MIR}}ou\frac{Red Edge-SWIR Cirrus}{Red Edge+SWIR Cirrus}$ |
| 15 | Optimized Soil Adjusted Vegetation Index | OSAVI | $(1+0,16)\frac{(Red Edge-Red)}{Red Edge+Red+0,16}$ |
| 16 | Ratio Vegetation Index | RVI | Red edge/Red |
| 17 | η ratio | VV/VH | Moreira et al., 2013 and Braun, (2021) |
| 18 | γ ratio | HH/VH | Veci (2016) and Braun, (2021) |
|  | ρ ratio | HH/VV | Moreira et al., 2013 and Braun, (2021) |
| 19 | Horizontal transmit and vertical polarization | VH | Veci (2016) and Braun, (2021) |
| 20 | Vertical transmit and vertical polarization | VV | Moreira et al., 2013 and Braun, (2021) |
| 21 | Horizontal transmit and horizontal receive polarization | HH | Veci (2016) and Braun, (2021) |
| 22 | Soil and Atmospherically Resistant Vegetation Index | SAVI | $\frac{Red Edge-Red}{Red Edge+Red+0,16}(1+L)$ |
| 23 | SLOPE | SLOPE | Using ArcGIS 10.7 |
| 24 | Transitions Performance Index | TPI | $Z- Ż$ Avec Z : l’altitude et Ż : l’altitude moyenne |
| 25 | Terrain Ruggedness Index | TRI | Using ArcGIS 10.7 |
| 26 | Transformed Vegetation Index | TVI | $\sqrt{\left( NDVI \right)+0,5}$ |
| 27 | Tasselled Cap – wetness | WET | 0,1509 Blue + 0,1973 Green + 0.3279 Red + 0,3406 Red Edge – 0,7112 SWIR1 – 0,4572 SWIR2 |


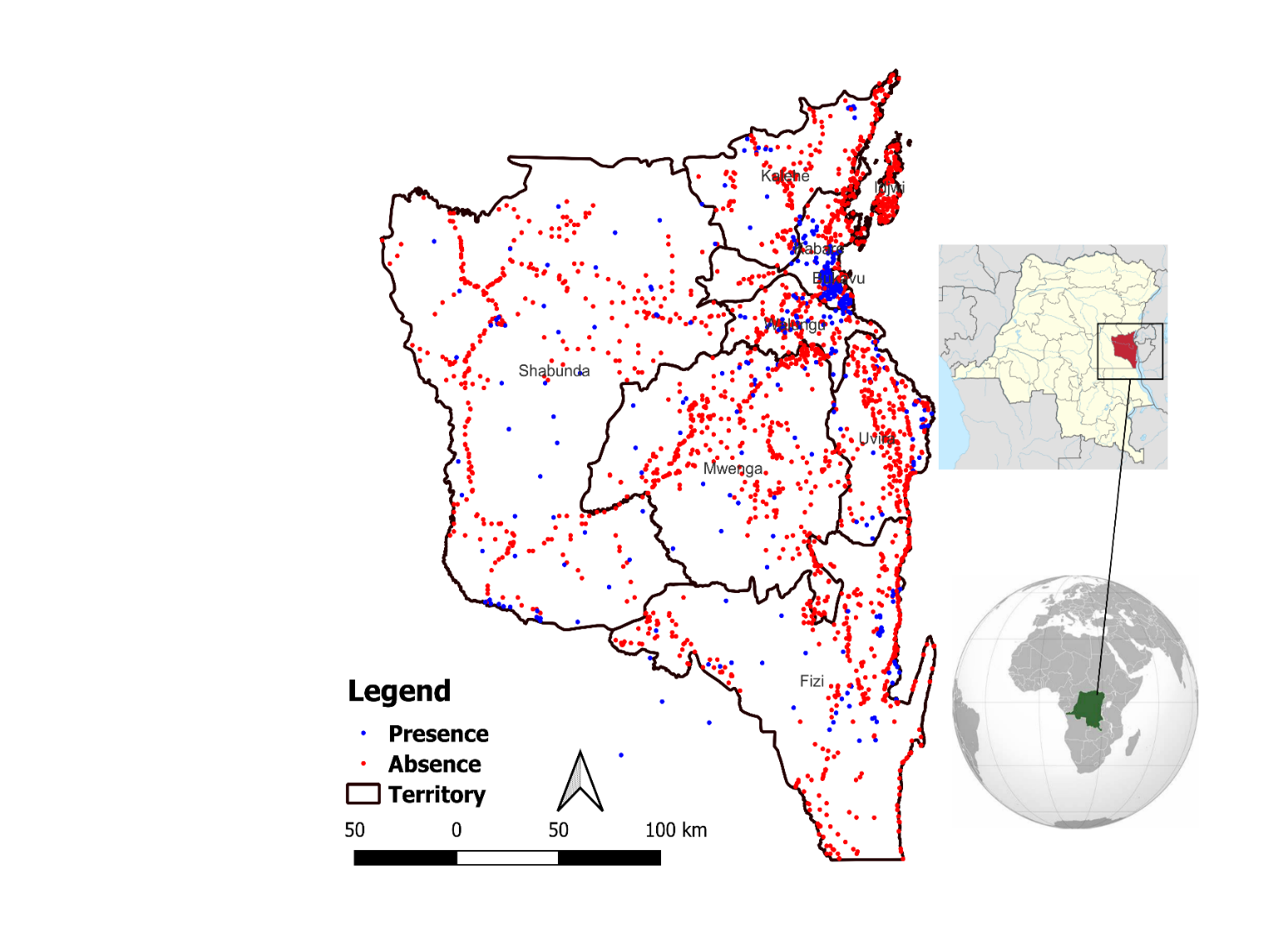


**Supplementary data S4: Presence and Absence data samples integrated in the model for wetland mapping (map created using ArcGIS 10.7 Esri-TM:** [**http://www.esri.com**](http://www.esri.com)**).**


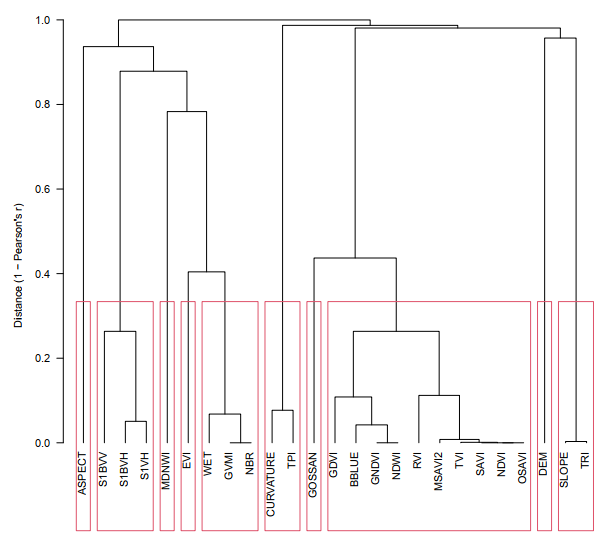


**Supplementary data S5: Variables selection before model integration using distance calculation (D= 1-Pearson’s r).** *Here only 25 variables were analyzed the two SAR image ratios (η and γ) were not integrated as they analyze the seasonality. VV, VH and HH were integrated.*


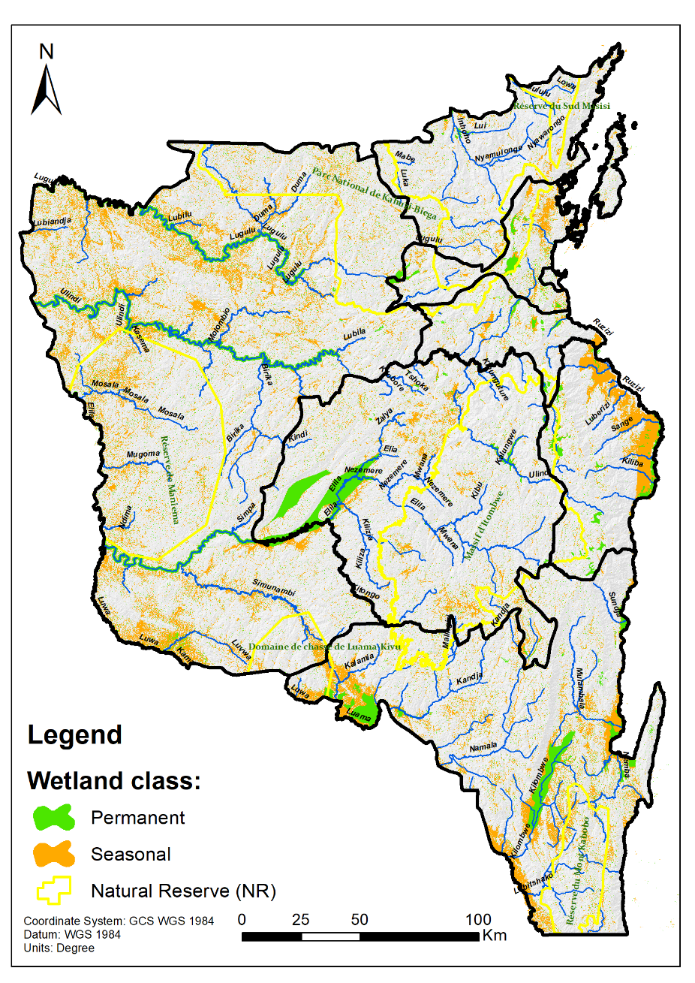


**Supplementary data S6. Wetland complex, main rivers and Natural reserves (NR) in the South-Kivu province (map created using ArcGIS 10.7 Esri-TM:** [**http://www.esri.com**](http://www.esri.com)**)**

***
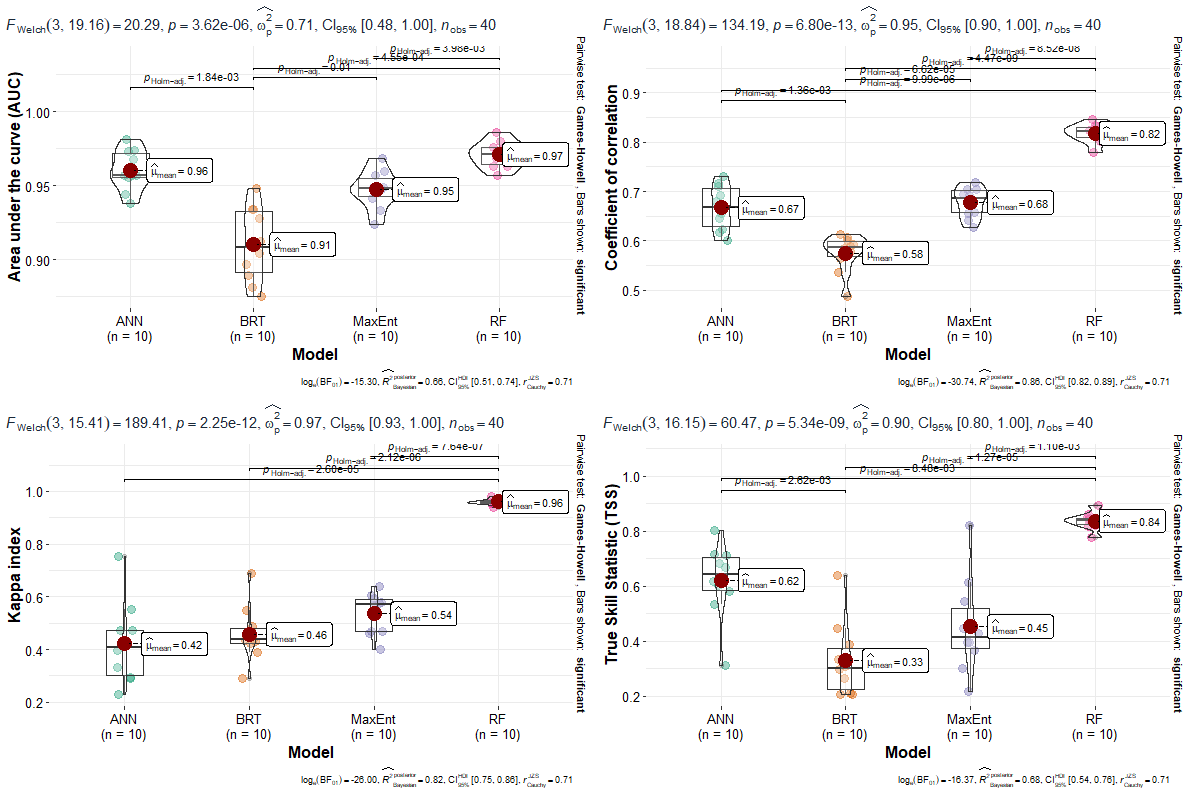
***

**Supplementary data S7. Model comparison using Delong test and Anova following the Welch test.**

******

******

**Supplementary data S8. Wetland and non-wetland surface (ha) and perimeter (km) in the three small territories (Walungu, Kalehe and Idjwi). *The two last images represent the data before and after log2 conversion.***

***
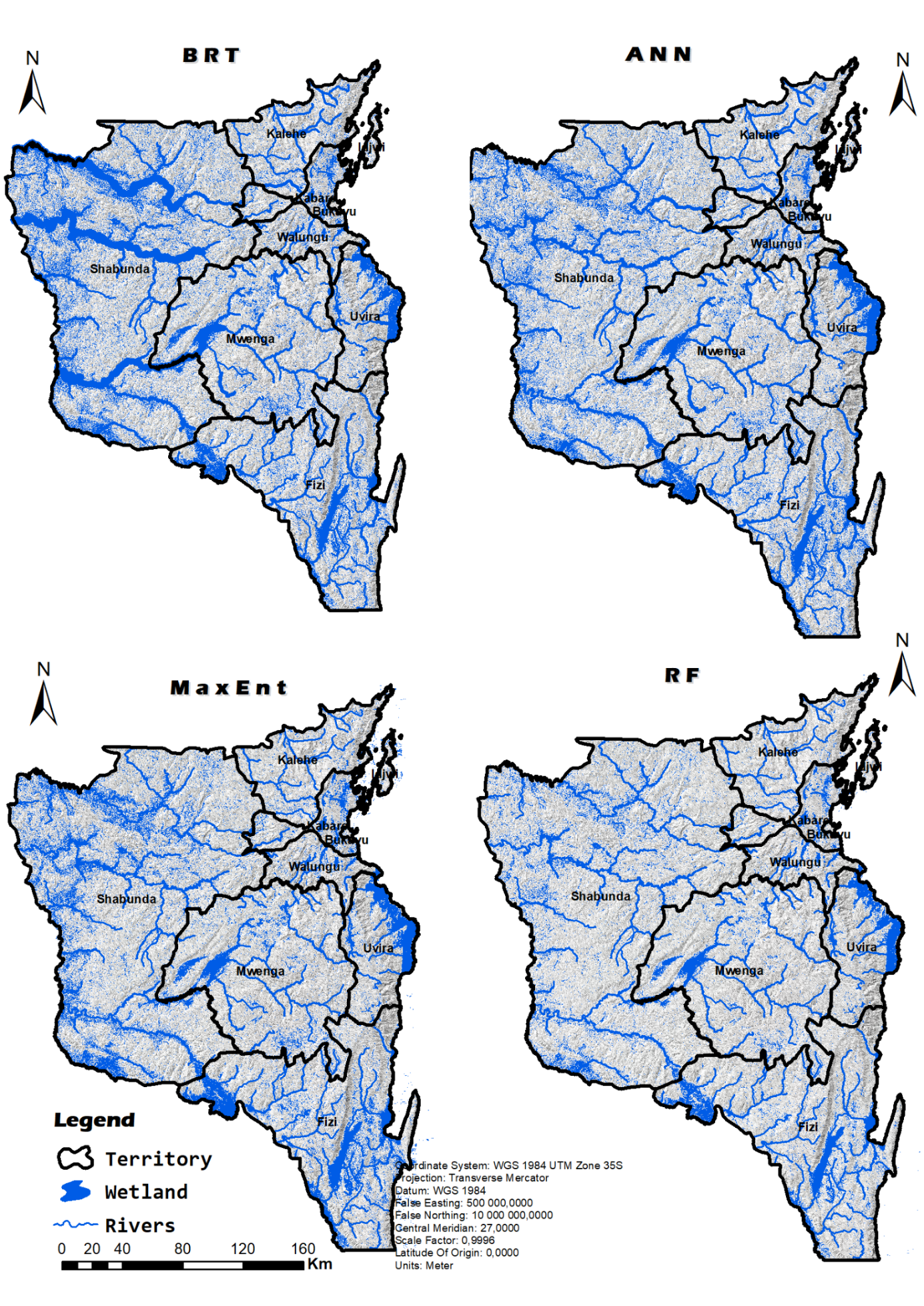
***

**Supplementary data S9. Potential distribution maps of wetland in South-Kivu province after reclassification (in two classes: blue: wetlands) the background is the hillshade produced using ALOS-DEM with transparency of 85% (map created using ArcGIS 10.7 Esri-TM:** [**http://www.esri.com**](http://www.esri.com)**)**


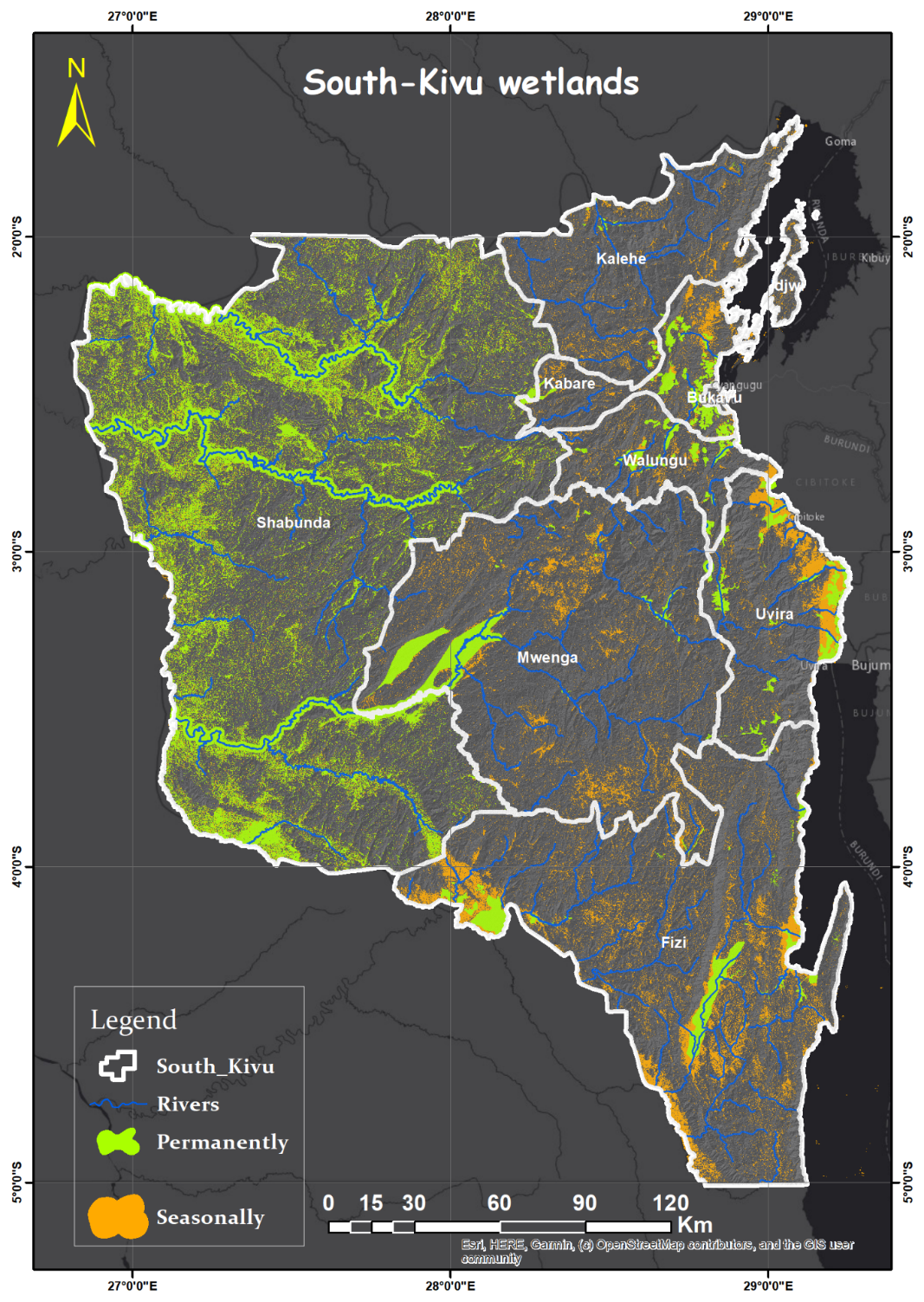


**Supplementary data S10. Permanent and seasonally flooded wetlands in the South-Kivu province (map created using ArcGIS 10.7 Esri-TM:** [**http://www.esri.com**](http://www.esri.com)**)**

**Supplementary data S11. Total surface of the territory and wetlands, and the proportion of the area for each territory of the South-Kivu province**

| Name | Surface (ha) | Wetland | Proportion (%) |
| --- | --- | --- | --- |
| Mwenga | 1,117,200 | 323,988 | 29 |
| Shabunda | 2,511,600 | 527,436 | 21 |
| Fizi | 1,578,800 | 473,640 | 30 |
| Kabare | 196,000 | 31,360 | 16 |
| Uvira | 314,800 | 59,812 | 19 |
| Walungu | 180,000 | 23,400 | 13 |
| Kalehe | 512,600 | 30,756 | 6 |
| Idjwi | 28,100 | 2,529 | 9 |


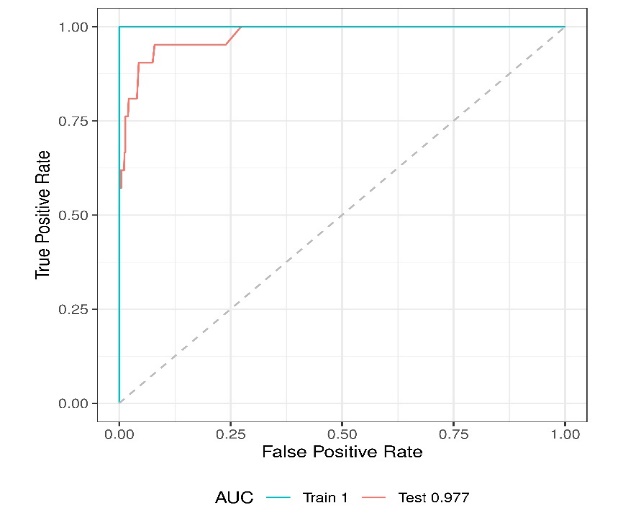

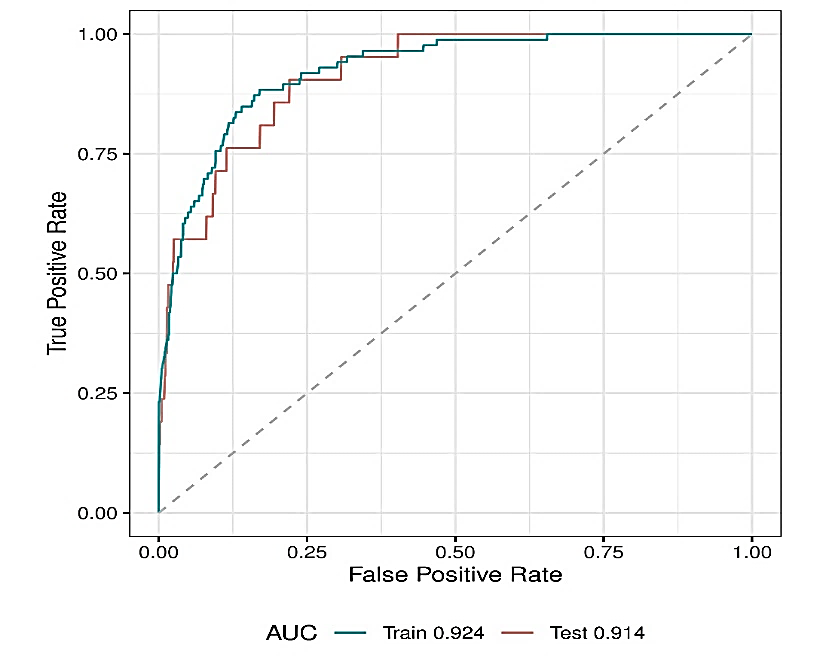


**Supplementary data S12. ROC AUC of RF without and with SAR data integration**

| 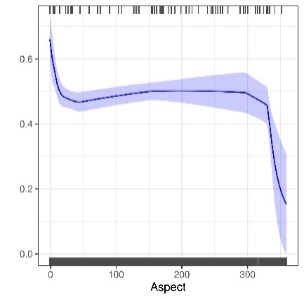 | 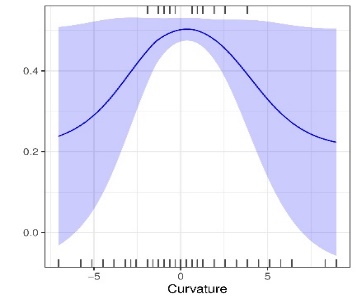 | 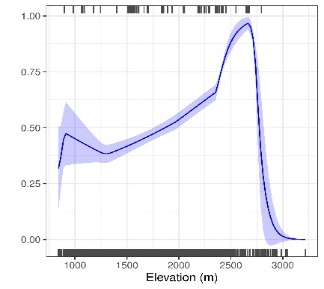 |
| --- | --- | --- |
| 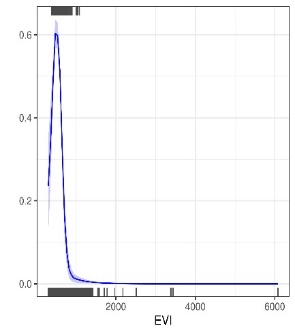 | 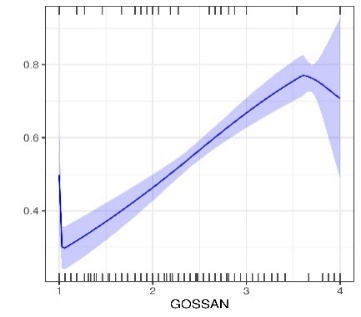 | 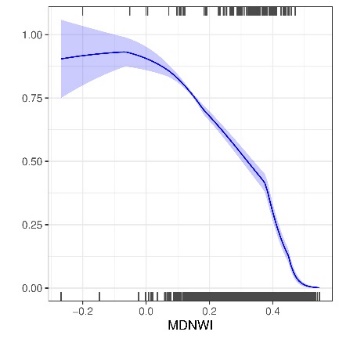 |
| 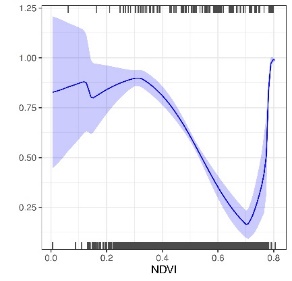 | 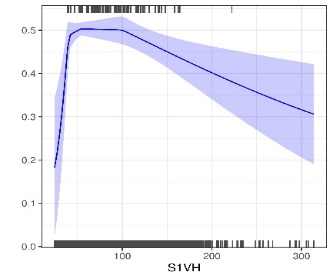 | 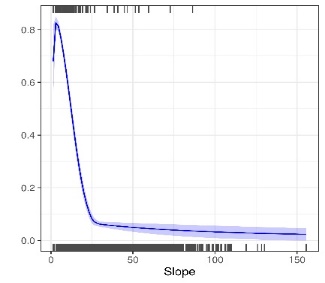 |
| 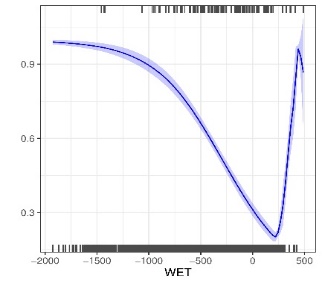 |  |  |

**Supplementary data S13. Function of partial dependence for each input variable to the RF model**

**Supplementary data 14. Strengths and weakness of models used.**

| **Model** | **Strength** | **Weakness** |
| --- | --- | --- |
| **BRT** | - Handles complex relationships and interaction between the input variables. - Has good predictive accuracy (due to its iterative learning process) - Handles multiple categorial and continuous predictors. - It is suitable to handle noisy and missing data (NA) | - Requires more time during training and intensive computation. - Prone to overfit if it is not properly tuned. - Sometimes not perform well with imbalanced datasets. |
| **RF** | - Robust and excellent predictive - Easy handles high-dimensional datasets - It is resistant to overfitting. - It provides measures of variable importance | - Difficulty in interpretation (as black-box model) - Requires more memory and computational resources. - Degradation of performance with irrelevant variables |
| **ANN** | - Helps to capture nonlinear relationships. - It is suitable for large and high-dimensional datasets. - Handles variety of data types - Possible to learn from data with minimal features engineering. | - Requires careful tuning of hyperparameters. - Prone overfitting (mainly of small datasets) - Sometimes not provide clear variable importance insights. - Time-consuming (mostly) |
| **Maxent** | - Suitable for species distribution modeling - Handles presence only data. - Handles both mix continuous and categorical predictors. - Provide probabilistic output | - Sensitive to bias in input data - Can produce unrealistic results. - Not handles well imbalanced datasets. - Prone to overfitting, especially with small sample size - Challenge in interpretation |
